# Phenotypic and molecular characterization of the claudin-low intrinsic subtype of breast cancer

**DOI:** 10.1186/bcr2635

**Published:** 2010-09-02

**Authors:** Aleix Prat, Joel S Parker, Olga Karginova, Cheng Fan, Chad Livasy, Jason I Herschkowitz, Xiaping He, Charles M Perou

**Affiliations:** 1 Lineberger Comprehensive Cancer Center, University of North Carolina, 450 West Drive, Chapel Hill, 27599, USA; 2 Department of Genetics, University of North Carolina, 450 West Drive, Chapel Hill, 27599, USA; 3 Department of Pathology & Laboratory Medicine, University of North Carolina, 450 West Drive, Chapel Hill, 27599, USA; 4 Department of Molecular & Cellular Biology, Baylor College of Medicine, One Baylor Plaza, Houston, 77030, USA

## Abstract

**Introduction:**

In breast cancer, gene expression analyses have defined five tumor subtypes (luminal A, luminal B, HER2-enriched, basal-like and claudin-low), each of which has unique biologic and prognostic features. Here, we comprehensively characterize the recently identified claudin-low tumor subtype.

**Methods:**

The clinical, pathological and biological features of claudin-low tumors were compared to the other tumor subtypes using an updated human tumor database and multiple independent data sets. These main features of claudin-low tumors were also evaluated in a panel of breast cancer cell lines and genetically engineered mouse models.

**Results:**

Claudin-low tumors are characterized by the low to absent expression of luminal differentiation markers, high enrichment for epithelial-to-mesenchymal transition markers, immune response genes and cancer stem cell-like features. Clinically, the majority of claudin-low tumors are poor prognosis estrogen receptor (ER)-negative, progesterone receptor (PR)-negative, and epidermal growth factor receptor 2 (HER2)-negative (triple negative) invasive ductal carcinomas with a high frequency of metaplastic and medullary differentiation. They also have a response rate to standard preoperative chemotherapy that is intermediate between that of basal-like and luminal tumors. Interestingly, we show that a group of highly utilized breast cancer cell lines, and several genetically engineered mouse models, express the claudin-low phenotype. Finally, we confirm that a prognostically relevant differentiation hierarchy exists across all breast cancers in which the claudin-low subtype most closely resembles the mammary epithelial stem cell.

**Conclusions:**

These results should help to improve our understanding of the biologic heterogeneity of breast cancer and provide tools for the further evaluation of the unique biology of claudin-low tumors and cell lines.

## Introduction

Genomic studies have established four major breast cancer intrinsic subtypes (luminal A, Luminal B, HER2-enriched, basal-like) and a normal breast-like group that show significant differences in incidence, survival and response to therapy [1-3]. However, as gene expression studies evolve, further subclassification of breast tumors into new molecular entities is expected to occur. In 2007, we identified a new molecular subtype, referred to as claudin-low, using 13 samples [5]. These distinct tumors were found in both human and murine breast tumor data sets and were characterized by the low gene expression of tight junction proteins claudin 3, 4 and 7 and E-cadherin, a calcium-dependent cell-cell adhesion glycoprotein. More recently, a tumor initiating cell (TIC) genomic signature derived from CD44^+^/CD24^-/low^-sorted cells and mammospheres obtained from primary human breast tumors was found to be exclusively enriched by gene expression in the claudin-low subtype [6,7], and the expression of this CD44^+^/CD24^-/low^/claudin-low profile increased in posttreatment samples after neoadjuvant chemotherapy or hormone therapy [7]. Overall, these studies suggest that the claudin-low tumor subtype lacks common epithelial cell features and is enriched for TIC features.

In this study, we comprehensively characterize the claudin-low subtype using an updated human tumor database and multiple independent data sets and present the pathological and chemotherapy response characteristics of this subtype of "triple negative" breast cancers. In contrast to the basal-like subtype, we provide evidence that claudin-low tumors are more enriched in epithelial-to-mesenchymal transition (EMT) features, immune system responses, and stem cell-associated biological processes. The molecular characterization of the claudin-low intrinsic subtype in tumors and cell lines reveals a breast cancer differentiation hierarchy that resembles the normal epithelial mammary developmental cascade.

## Materials and methods

### Human breast tumor microarray data sets

All human tumor and normal tissue samples were collected using Institutional Review Board (IRB)-approved protocols and were obtained from fresh frozen invasive breast carcinomas that were profiled as described previously using oligo microarrays (Agilent Technologies, Santa Clara, CA, USA) [8]; we used all the microarrays from Herschkowitz *et al. *[5], Parker *et al. *[9] and Hennessy *et al. *[6], plus 39 new additional samples presented here. All microarray and patient clinical data are available in the University of North Carolina (UNC) Microarray Database [10] and have been deposited in the Gene Expression Omnibus (GEO) under the accession number GEO:GSE18229 (referred to here as the UNC337 set). The probes or genes for all analyses were filtered by requiring the lowest normalized intensity values in both sample and control to be > 10. The normalized log_2 _ratios (Cy5 sample/Cy3 control) of probes mapping to the same gene (Entrez ID as defined by the manufacturer) were averaged to generate independent expression estimates. In the resulting UNC337 matrix, no significant batch effects were observed. We also used publicly available microarray and patient clinical data from the following breast cancer data sets: NKI295 [11,12], MDACC133 [13] and NKI113 [14]. In MDACC133, raw data were normalized using the robust multiarray analysis (RMA) normalization approach. In all data sets, genes were median-centered within each data set and samples were standardized to zero mean and unit variance before other analyses were performed.

### Gene expression signatures

We analyzed the mean expression of multiple previously published gene signatures [7,15-19]. Briefly, these signatures include leukocyte-related signatures from Palmer *et al. *[17]: CD8 (*n *= 10 genes), B_Cell (*n *= 286), T_Cell (*n *= 178), and GRANS (*n *= 353). Stromal-related signatures were obtained from West *et al. *[16] (*n *= 402; DTF and SFT signatures combined) and Beck *et al. *[19] (*n *= 174). Genes enriched more than twofold in mammosphere-derived cells compared with differentiated cells were obtained from Dontu *et al. *[15] (*n *= 58). From Shipitsin *et al. *[18], we calculated the mean expression of the upregulated (*n *= 357) and downregulated (*n *= 353) genes from CD44^+ ^versus CD24^+ ^breast cancer cells from metastatic pleural effusions. From Creighton *et al. *[7], we calculated the mean expression of the CD44^+^/CD24^-/low^/mammosphere signature reported (*n *= 119 upregulated genes; *n *= 279 downregulated genes). Finally, the proliferation and luminal gene cluster signatures were hand-picked (node correlation > 0.75) from the unsupervised intrinsic hierarchical clustering of the UNC337 using the intrinsic list of Parker *et al. *[9] and average linkage clustering using Cluster v2.12 (M. Eisen) [20] as shown in Figure S1 in Additional file 1. Gene lists from all genomic signatures are displayed in Supplemental Data in Additional file 2.

### Breast cancer cell line microarray data set

We analyzed a data set that included Affymetrix U133A gene expression microarrays of 52 breast cancer cell lines [21]. Raw data were normalized using RMA, and genes were median-centered before analyses. Among the 52 breast cancer cell lines analyzed, DU4475, HCC1008 and HCC1599 cell lines were not included in Neve *et al. *[21].

### Mouse breast tumor microarray data set

All mouse samples from the UNC were collected from fresh frozen invasive breast carcinomas as described previously [5] using Agilent mouse oligo microarrays. Data normalization and preprocessing were identical to that described for the UNC337 data set. We used only samples (*n *= 104) from our previous publication that were included in 1 of the 10 mouse classes [5].

### Claudin-low and normal breast Euclidian centroid-based predictors

To robustly identify claudin-low samples, we built two predictors on the basis of either our human tumor data or the cell line data of Neve *et al. *[21]. To build a predictor, we first selected those genes that were significantly differentially expressed between claudin-low tumors defined by SigClust [22] (or cell lines) and all other subtypes using a two-class, unpaired SAM, with < 5% false discovery rate (FDR). Then we used these gene lists and calculated a claudin-low centroid and an "others" centroid from the training data. For every sample, we calculated the Euclidean distances to the two centroids and assigned the class of the nearest centroid. Using the same methodology, we also built a normal breast predictor by selecting those genes that were significantly differentially expressed between normal breast tissues and breast tumors using a two-class, unpaired SAM, with 0% FDR. Note that these gene lists are also included in Supplemental Data in Additional file 2.

### Intrinsic subtype classification

For all human breast tumor studies, intrinsic subtype classification was performed using the PAM50 predictor [9]. Human claudin-low tumor samples were identified using either SigClust [22] or the nine-cell line claudin-low predictor. Samples identified by SigClust [22] or the nine-cell line claudin-low predictor were called claudin-low, regardless of the PAM50 call. For breast cancer cell lines, the claudin-low subtype classification was based on unsupervised hierarchical clustering using the intrinsic list of Parker *et al. *[9] and the node identified in Figure S4 in Additional file 1. The complete gene list of the nine-cell line claudin-low predictor can be found in Supplemental Data in Additional file 2.

### Mammary developmental analyses

Public data sets from Raouf *et al. *[23] and Lim *et al. *[24] were downloaded from GEO and assigned NCBI Entrez gene identifiers as available in GEO. Samples were scaled to mean zero and variance of 1. Features were then collapsed to the mean of each gene identifiers. In Lim *et al. *[24], three epithelial cell-enriched subpopulations were profiled on DNA microarrays: mammary stem cells (MaSC), luminal progenitors (pL) and mature luminal cells (mL). We created a differentiation predictor for each sample as a measure of any sample's position along a MaSC → pL → mL axis as defined by gene expression. Distance-weighted discrimination (DWD) was used to determine the direction of greatest variation from MaSC to pL and pL to mL. To map a sample onto this axis of differentiation, the pL centroid was set as the origin, and the MaSC and mL centroids were transformed to length 1 (sum of squares equals 1) to map a sample onto this axis of differentiation. Before mapping a sample onto this axis, it is assumed that the test data set covers the range of differentiation, which allows median centering of genes to correct for platform bias. Test samples are then adjusted using the parameters for placing pL at the origin and are transformed to length 1. Each sample is then projected onto the MaSC → pL axis and the pL → mL axis by calculating the inner product of the sample and the MaSC or mL vectors identified by DWD. The difference of the two projected positions of each sample along the MaSC → pL → mL axis is referred to as the differentiation score. In the UNC microarray database web site [10], we have provided the detailed R commands and files regarding the differentiation predictor.

### Mammospheres from normal breast tissues

Fourteen normal breast tissues were dissociated mechanically and enzymatically as described in Stingl *et al. *[25]. The samples were procured and used according to approved IRB protocols for research in human subjects. Mammospheres were cultured according to Dontu *et al. *[15], and single cells were plated onto ultralow attachment plates (Corning) at a density of 20,000 viable cells/ml. RNA was purified using RNeasy Mini kit (Qiagen) after 14 to 20 days in primary culture (first passage), and microarrays were performed as described above.

### Immunohistochemistry

Formalin-fixed, paraffin-embedded tissue sections (~5 μm thick) were processed using standard immunohistochemistry methods as previously described [26]. The sections were incubated for 60 min at room temperature with primary antibody to claudin 3 (dilution 1:100; Invitrogen, Catalog No. 18-7340) or E-cadherin (Clone No. ECH-6, pre-diluted; Cell Marque). The slides were incubated for 45 min with diluted biotinylated secondary antibody (1:250 dilution) for 30 min with Vectastain Elite ABC reagent (Vector Laboratories). Sections were incubated in peroxidase substrate solution for visualization. Slides were counterstained with hematoxylin and examined by light microscopy. Tumor immunoreactivity was scored in a blinded fashion by two investigators (JIH and XH) into two categories: negative/weak positive and moderate/strong positive.

### Immunofluorescence

Formalin-fixed, paraffin-embedded sections (~5 μm thick) were processed using standard immunostaining methods as previously described [5]. The primary antibodies and their dilution were vimentin (mouse anti-vimentin IgG1-κ, dilution 1:100; Invitrogen, Catalog No. 18-0052), keratin 5 (rabbit anti-human, dilution 1:500; Abcam, Catalog No. ab24647), and keratin 19 (Abcam, Catalog No. ab7754, mouse anti-human IgG2a; dilution 1:200). Secondary antibodies for immunofluorescence were conjugated with Alexa Fluor-568 (Red, keratin 5 and 19) or Alexa Fluor-488 (Green, vimentin) fluorophores (1:200; Molecular Probes, Invitrogen). Dual positivity was scored in a blinded fashion by XH into two categories: negative meaning no dual positive cells and positive meaning the presence of dual positive cells.

### Cell lines

SUM159PT cells (Asterand) were maintained in Ham's F-12 medium with 5% fetal bovine serum (FBS), insulin (5 μg/ml), and hydrocortisone (1 μg/ml). MCF-7 was cultured in RPMI with 10% FBS [27], and SUM149PT was maintained in HuMEC media with supplements (Gibco) with and without 5% FBS [28]. All cell lines were grown at 37°C and 5% carbon dioxide.

### Fluorescence-activated cell sorting (FACS) and microarray analysis

Nonconfluent cell cultures were trypsinized and filtered to produce single cell suspension, counted, washed with Hanks' balanced salt solution (Stem Cell Technologies) containing 2% FBS and stained with antibodies specific for human cell surface markers: EPCAM-fluorescein isothiocyanate (Stem Cell Technologies) and CD49f-phycoerythrin (BD Pharmingen). A total of 500,000 cells were incubated with antibodies for 30 min at 4°C. Cells were washed from unbound antibodies and immediately analyzed using Beckman-Coulter (Dako) CyAn ADP or sorted using BD FACScan. RNA was purified from sorted cells using the RNeasy Mini kit, and microarrays were performed as described above.

### Statistical analyses

All microarray cluster analyses were displayed using Java Treeview version 1.1.3. Average linkage hierarchical clustering was performed using Cluster v2.12 [20]. Biologic analysis of microarray data was performed with the DAVID annotation tool [29]. ANOVA and Student's *t*-tests for gene expression data, Fisher's exact test for neoadjuvant clinical data, χ^2 ^tests for pathological data, and the Cox model were performed using R [30]. Survival curves were calculated by the Kaplan-Meier method and compared by the log-rank test using WinStat v2007.1. Reported *P *values are two-sided.

## Results

### Molecular characterization of the claudin-low breast tumor subtype

**Figure 1:**
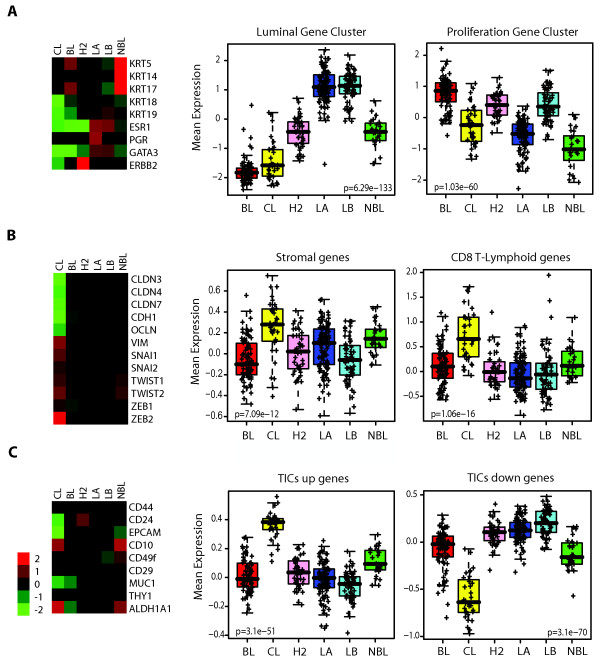
**Average expression of important genes and gene signatures across the intrinsic breast cancer subtypes**. **(a) **Classical markers used to characterize breast tumors are shown for mRNA expression levels for basal markers (keratins 5 [KRT5], 14 [KRT14] and 17 [KRT17]), luminal markers (keratins 18 [KRT18] and 19 [KRT19]), ER (ESR1), PR, GATA3 and HER2 (ERBB2). *Right*: Box-and-whisker plot for expression of the luminal and proliferation gene signatures. **(b) **Markers of EMT (vimentin [VIM], Snail-1 [SNAI1], Snail-2 [SNAI2], TWIST1, TWIST2, ZEB1, ZEB2, E-cadherin [CDH1], and claudins 3 [CLDN3], 4 [CLDN4] and 7 [CLDN7]). *Right*: expression of stromal- and immune-related signatures [16,17]. **(c) **Markers of stem cells/TICs/epithelial differentiation (CD44, CD24, EpCAM, CD10, CD49f, CD29, MUC1, THY1, and ALDH1A1). *Right*: Previously published stem cell-like signature [7]. Each colored square on the left side represents the relative mean transcript abundance (in log_2 _space) for each subtype with highest expression being red, average expression being black, and lowest expression being green. BL, basal-like; CL, claudin-low defined by SigClust [22]; H2, HER2-enriched; LA, luminal A; LB, luminal B; NBL, normal breast-like. *P *values shown were calculated by comparing gene expression means across all subtypes.

To identify the molecular characteristics of claudin-low tumors, we created a large genomic data set by combining three of our previously published data sets [5,6,9] and included 37 new tumor samples (*n *= 337; UNC337, GEO series GSE18229). Hierarchical clustering analysis of this data set using the ~1,900 intrinsic gene list of Parker *et al. *[9] identified the major intrinsic molecular subtypes, including the claudin-low subtype (Figure S1 in Additional file 1). The validity of the claudin-low sample cluster was confirmed by parsing the dendogram with SigClust [22] (*P *< 0.001); notably, this clustering analysis placed the claudin-low tumors in close proximity to the basal-like subtype and was composed of 32 arrays, representing 32 patients (~12% of all patients). Compared to the luminal A, luminal B, HER2-enriched, and basal-like subtypes, claudin-low tumors showed inconsistent expression of basal keratins (keratins 5, 14 and 17) and low expression of HER2 and luminal markers such as ER, PR, GATA3, keratins 18 and 19 and the luminal gene cluster (Figure 1a). Despite the apparent similarity to basal-like tumors, claudin-low tumors as a group did not show high expression of proliferation genes and thus are likely slower-cycling tumors. Indeed, significantly lower messenger RNA (mRNA) expression of the cell cycle gene Ki67 was observed in claudin-low tumors when compared with basal-like tumors (*P *< 0.0001, Student's *t*-test; Figure S2 and Table S1 in Additional file 1).

To better characterize the claudin-low molecular subtype, we identified those genes differentially expressed in claudin-low tumors compared with other tumors or subtypes. We found 1,308 and 359 genes significantly higher and lower in expression in claudin-low tumors, respectively (Table S2 in Additional file 1). Overall, claudin-low tumors highly expressed genes involved in immune system response (i.e. CD79b, CD14 and vav1), cell communication (chemokine [C-X-C motif] ligand 12), extracellular matrix (vimentin, fibroblast growth factor 7), cell differentiation (Krüppel-like factor 2, interleukin 6), cell migration (integrin a5, moesin) and angiogenesis (vascular endothelial growth factor C, matrix metallopeptidase 9) [29]. Conversely, expression of various epithelial cell-cell adhesion genes such as claudin 3, claudin 4, claudin 7, occludin and E-cadherin was significantly lower as previously reported [5] (Figure 1b). Further immunohistochemical analysis of 103 breast tumors of the UNC337 data set revealed that compared to the basal-like subtype, the claudin-low tumor subtype had a preponderance for low to absent expression of E-cadherin and claudin 3 (45% vs. 11% for E-cadherin, *P *< 0.05; 59% vs. 11% for claudin 3, *P *< 0.005; χ^2 ^test). Similarly, when compared to all other tumors (basal-like, HER2-enriched, luminal A, luminal B and normal breast-like) as a single group, the claudin-low tumor subtype maintained its characteristic for low to absent expression of E-cadherin and claudin 3 (45% vs. 15% for E-cadherin, *P *< 0.005; 59% vs. 22% for claudin 3, *P *< 0.001; χ^2 ^test) (Figure S3 in Additional file 1).

Concordant with the expression of markers of mesenchyme and immunity, we observed high expression of stromal-specific and lymphocyte- or granulocyte-specific gene signatures in claudin-low tumors compared to the other intrinsic subtypes [16,17,19] (Figure 1b, Figure S2 in Additional file 1). These findings, together with the low expression of epithelial cell-cell adhesion molecules such as E-cadherin, are consistent with an EMT (changes in cell phenotype between epithelial and mesenchymal states) [31] in claudin-low tumors and a potential recruitment of multiple types of leukocytes into these tumors.

We next explored the mRNA expression of the TIC gene markers CD44 and CD24 and cell surface markers of epithelial differentiation such as MUC1, CD49f, and epithelial cell adhesion molecule (EpCAM) across the intrinsic subtypes (basal-like, claudin-low, luminal A, luminal B, HER2-enriched) and the normal breast-like group to determine their differentiation status. Overall, claudin-low tumors showed low mRNA expression of differentiated luminal cell surface markers (CD24, EpCAM and MUC1), while markers CD44 and CD49f were higher when compared to differentiated luminal tumors (*P *< 0.05, Student's *t*-test; Figure 1c, Figure S2 in Additional file 1). The expression pattern of these gene markers is concordant with CD44^+^/CD24^-/low ^and CD49f^+^/EpCAM^-/low ^antigenic phenotypes, which have been previously shown to be enriched in breast TICs [32,33] and mammary stem cells (MaSCs) [24]. Second, we observed that claudin-low tumors compared to the other tumor subtypes, except for the normal breast-like group, showed the highest mRNA expression of ALDH1A1, which has recently been proposed to be another marker of breast stem cells and TICs [34] but also noted in stromal cells [24,34,35]. Conversely, basal-like tumors did not show significantly lower expression of CD24 as a group, nor did they show high mRNA expression of ALDH1A1 (Figure 1b and Figure S2 in Additional file 1). This contrasts with other studies that have linked the basal-like subtype with stem cell- or embryonic cell-like features [36,37]; however, these other studies did not examine claudin-low tumors as a separate group, and in the absence of a claudin-low predictor, claudin-low tumors are typically classified as basal-like (or normal breast-like) by the PAM50 gene expression assay [9].

To further explore the potential enrichment for breast stem cells and TIC features, we evaluated the expression of three breast stem cell-like signatures [7,15,18] across the different subtypes. All signatures were highly enriched (*P *< 0.0001, Student's *t*-test; Supplementary Material in Additional file 2) in the claudin-low subtype despite the various derivations used of each signature (Figure 1c, Figure S2 in Additional file 1). Interestingly, these three stem cell-like signatures were representative of distinct gene expression subsets, among which < 10% of the genes overlapped. These data suggest that different biological processes associated with TICs converge in the claudin-low tumor subtype.

### Identification of the claudin-low profile in a panel of breast cancer cell lines

**Figure 2:**
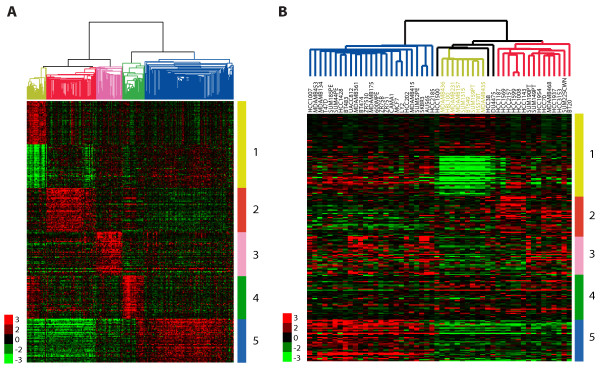
**Identification of the claudin-low subtype in a panel of breast cancer cell lines**. Gene clusters that characterize each primary human tumor subtype are shown in the human and cell line gene expression data sets. In both data sets, array trees have been derived by unsupervised hierarchical clustering using the intrinsic list from Parker *et al. *[9] as shown in Figure S1A and S4A in Additional file 1. **(a) **The top 50 upregulated genes associated with each molecular subtype, including the top 50 downregulated genes in claudin-low tumors, are shown in the UNC337 database. Top genes were selected after performing a two-class SAM (FDR = 0%) between each molecular subtype versus others. Luminal A and B subtypes were combined into the luminal subtype. In the tree, the yellow node denotes the claudin-low tumors defined by SigClust [22]. **(b) **Gene clusters characteristic of each tumor molecular subtype are shown in 52 breast cancer cell lines from Neve *et al. *[21]. Missing genes have been omitted. In the tree, the yellow node denotes the most highly correlated cell lines that best resemble the claudin-low subtype. 1 (yellow), claudin-low gene cluster of upregulated and downregulated genes; 2 (red), basal-like gene cluster; 3 (pink), HER2-enriched gene cluster; 4 (green), normal breast-like gene cluster; 5 (blue), luminal gene cluster.

To investigate if potential *in vitro *counterparts for these tumors exist, we analyzed a data set of 52 breast cancer cell lines [21] by hierarchical cluster analysis using our most recent human breast tumor intrinsic classification list [9]. The three major subgroups (luminal, basal B and basal A) identified previously by Neve *et al. *[21] were evident, with nine basal B cell lines clustering together with a node correlation of 0.59 (Figure S4 in Additional file 1). These cell lines showed low expression of the ER, HER2 and claudin 3, claudin 4 and claudin 7 (Figure S4 in Additional file 1). We identified those genes whose expression distinguished each human tumor subtype using significance analysis of microarrays (SAM) in our UNC337 tumor database, including a list defining the normal breast-like group (Figure 2a). These nine cell lines (MDA-MB-435, MDA-MB-436, Hs578T, BT549, MDA-MB-231, MDA-MB-157, SUM1315MO2, SUM159PT and HBL100) showed a gene expression pattern similar to the claudin-low tumor subtype with the lowest expression of genes involved in epithelial cell-cell adhesion (i.e., E-cadherin and claudins 3, 4 and 7), luminal differentiation (i.e., CD24, EpCAM) and high values for the CD44/CD24 and CD49f/EpCAM mRNA ratios (Figure S4, Table S3 in Additional file 1). To complement these clustering analyses, we developed a claudin-low centroid-based predictor using the UNC337 tumor data set and the SigClust-defined claudin-low group versus all others, and objectively classified the 52 cell lines as claudin-low or not; as expected, the human tumor-based claudin-low predictor identified these nine cell lines as claudin-low (Figure S5 in Additional file 1).

The gene cluster that identifies the *in vivo *defined normal breast-like group was not differentially expressed by any of the 52 cell lines, suggesting overall that none of these cell lines show a normal breast-like phenotype (Figure 2b). The normal breast-like group of primary specimens is usually composed of actual normal breast samples and a small number of primary tumors [9,38], the latter of which we believe show the high expression of true normal tissue genes due to significant normal breast contamination [9]. In cell culture, however, there is no such contamination by normal epithelial or nonepithelial cells, and thus this lack of contamination may explain why normal breast-like tumor cell lines are not identified as such by the clustering. To be more objective, we applied a normal breast centroid-based predictor (normal breast versus all tumors using the UNC337 database) to the cell line data, and no cell line was classified as normal breast (Figure S5 in Additional file 1). Contamination with other tissues or cells is not uncommon in other tumor subtypes [1]. For example, within the top highly expressed genes in claudin-low tumors versus all others, there are several wound response-related genes (i.e., CD28, CD52) that are not expressed by the breast cancer cell lines (Figure 2b), potentially due to significant immune or stromal cell content in the tumor. Overall, these data suggest that the nine previously described breast cancer cell lines are most similar to claudin-low tumors in *in vivo *specimens.

### Building a Cell-line claudin-low centroid-based predictor

Because primary human claudin-low tumors are highly enriched for immune system genes (and lymphocytes) when supervised analyses are performed, the expression of nonepithelial cell genes would likely increase the false positivity of the human claudin-low predictor when applied to other tumor data sets, because, for instance, tumors would be called claudin-low or not based upon the high expression of immune cell genes. Therefore, we developed a claudin-low centroid-based predictor using the cell line database of Neve *et al. *[21]. First, we evaluated its accuracy by applying the predictor onto our human tumor database (UNC337). The nine-cell line claudin-low predictor identified 37 samples (~11%) as claudin-low, including the 28 claudin-low samples previously identified by hierarchical clustering. The remaining nine samples identified by the predictor were previously called basal-like (*n *= 7), normal breast-like (*n *= 1) and HER2-enriched (*n *= 1) by the PAM50 predictor [9]. Overall, the nine-cell line claudin-low predictor showed 87.5% sensitivity, 97.0% specificity, and 75.7% and 98.7% positive and negative predictive values, respectively, if the SigClust-defined claudin-low group is considered the gold standard.

We then used the nine-cell line claudin-low predictor to identify this subtype in a mouse tumor database [5], which represents 13 different mouse models grouped in 10 different classes on the basis of their gene expression profiles (Groups I to X). Interestingly, all claudin-low samples identified by the centroid predictor (*n *= 9) were included in Group II, which we previously highlighted for its mesenchymal features (i.e., spindle-shaped cells). These nine murine claudin-low tumor samples were derived from six different mouse models (Brca1Co/Co;TgMMTV-Cre;p53+/-, DMBA-induced, p53-/- transplant, p53+/- IR, TgMMTV-Neu and TgWAP-T121) and overall showed similar enrichment for EMT markers and human mesenchymal and stem cell-like signatures, including decreased expression of proliferative- and luminal-associated genes (Figure S6 in Additional file 1). No murine normal mammary tissue sample was classified as claudin-low. These analyses suggest that a cell line centroid-based approach, by focusing on genes expressed in epithelial cells only, might give more accurate classification of tumors, which could be evaluated in future studies focused on tumor subtyping.

### Clinical-pathological characteristics of claudin-low breast tumors

**Figure 3:**
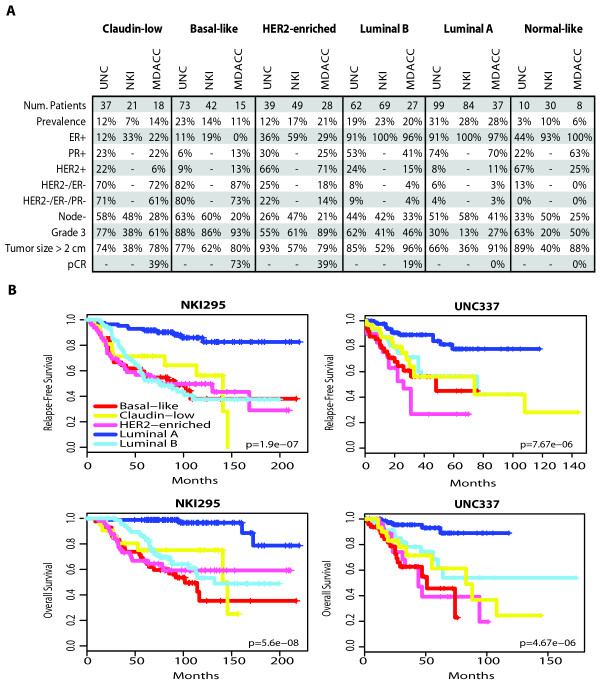
**Clinical and pathological characteristics and prognosis of all intrinsic subtypes, including claudin-low tumors, across three independent breast cancer data sets**. **(a) **Percentages of the different clinical-pathological characteristics in the UNC337 data set and two publicly available data sets (NKI295 and MDACC133). ER/PR/HER2 scores of the UNC337 database were based on clinically validated methods. **(b) **Survival data of the different molecular subtypes are shown for the UNC337 database and NKI295. Normal breast-like samples have been removed from this analysis. The UNC337 set represents a heterogeneously treated group of patients treated in accord with the biomarker status, whereas NKI295 is predominantly a local therapy only cohort.

To determine for the first time the clinical-pathological characteristics of human claudin-low breast tumors, we evaluated our breast cancer patient database (UNC337) and two independent gene expression data sets (NKI295 and MDACC133) [11-13] using the nine-cell line claudin-low predictor and the previously published PAM50 subtype predictor (Figure 3a) because these two objective centroid predictors have been demonstrated to be the most robust to classify breast tumors into discrete subtypes. Across all three databases, claudin-low tumors showed a prevalence of 7 to 14%, and were mostly ER-/PR-/HER2- (also known as triple-negative tumors, 61 to 71%). Conversely, the majority of triple-negative tumors were either basal-like (39 to 54%) or claudin-low (25 to 39%), followed by HER2-enriched (7 to 14%), luminal B (4 to 7%), luminal A (4 to 5%) and normal breast-like (1%). In terms of prognosis, Kaplan-Meier survival analysis revealed that claudin-low tumors have a worse prognosis compared to luminal A tumors in both the UNC337 (hazard ratio [HR] of 2.83 and 5.66 for relapse-free survival [RFS] and overall survival [OS], respectively; *P *< 0.05) and NKI295 data sets (HR of 4.71 and 17.98 for RFS and OS, respectively; *P *< 0.001). Conversely, similar survival curves were observed between claudin-low tumors and the other poor prognosis subtypes such as luminal B, HER2-enriched and basal-like tumors (Figure 3b). Normal breast-like samples were omitted from survival analyses because they are likely significantly contaminated by normal breast tissue, and thus their true tumor biology is masked.

We also evaluated the potential association between claudin-low tumors and treatment response by using the MDACC breast cancer patient data set (133 pretreated samples) of tumors treated with neoadjuvant anthracycline/taxane-based chemotherapy [13]. Notably, claudin-low tumors showed a lower pathological complete response (pCR) rate after anthracycline/taxane-based chemotherapy compared to basal-like tumors (38.9% vs. 73.3% pCR rates; *P *= 0.08, Fisher's exact test), but their pCR rate was higher than luminal A or B tumors; interestingly, the apparent pCR rate of the basal-like tumors increased from 59% (reported in Parker *et al. *[9]) to ~73% when the claudin-low subtype was included, because among 18 claudin-low tumors identified in this set, 12 (67%) of 18 of them were previously identified as basal-like and the response rate of this subgroup of patients was 41.7%. These findings suggest that claudin-low tumors show some chemotherapy sensitivity but overall have a poor prognosis and may not be managed effectively with existing chemotherapy regimens.

Twenty-one claudin-low samples were examined histologically by a pathologist (CL; Table S4 in Additional file 1) to further clinically characterize claudin-low tumors. Among the samples evaluated, 9 (43%) of 21 had noteworthy histological differentiation patterns, including medullary-like features (5/21, 24%) such as pushing margins and brisk tumor lymphocytic infiltration, two samples (2 of 21, 10%) showed metaplastic differentiation, one sample showed mixed ductal/lobular features, and one sample was a pure micropapillary carcinoma. The remaining 12 samples (12 of 21, 57%) were invasive ductal carcinomas not otherwise specified. Overall, lymphoid infiltration was evident in a total of seven samples (total of 7 of 19, 37%), which might explain the high mRNA expression of immune response genes present in these tumors. Among the other claudin-low samples that could not be examined histologically (*n *= 16), 50% (8/16) had a previous diagnosis of metaplastic tumors. It is interesting to note that two claudin-low cell lines, Hs578T and MDA-MB-157, were derived from metaplastic [39] and medullary [40] carcinomas.

**Figure 4:**
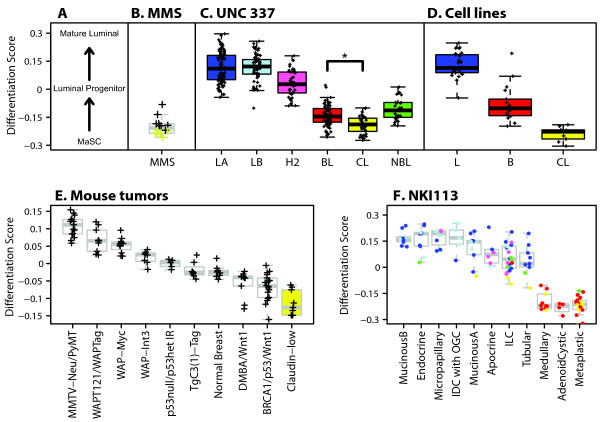
**Epithelial differentiation score analysis of normal mammary tissue, human breast tumors, human cell lines and mouse mammary tumors**. **(a) **Differentiation axis based on Lim *et al. *[24] data. **(b) **Mammospheres (MMS; *n *= 14) derived from normal breast tissue. Yellow crosses identify claudin-low MMS (*n *= 6, 43%) as defined by the nine-cell line claudin-low predictor. **(c) **Tumors and the normal breast-like group from the UNC337 database. **(d) **Breast cancer cell lines. Except for the nine claudin-low cell lines, we used the subtype calls (luminal [L] and basal [B]) as reported in Neve *et al. *[21]. **(e) **Mouse tumors from Herschkowitz *et al. *[5]. **(f) **Histological special types of breast cancer obtained from the NKI113 database [14]. Colored dots or boxes denote the subtype cells. IDC with OGC, invasive ductal carcinoma with osteoclastic giant cells; ILC, invasive lobular carcinoma; BL (red), basal-like; CL (yellow), claudin-low defined by the nine-cell line claudin-low predictor; H2 (pink), HER2-enriched; LA (dark blue), luminal A; LB (light blue), luminal B; NBL (green), normal breast-like. *P < 0.0001.

Since the pathological examination of claudin-low tumors identified special histological features, we applied the nine-cell line claudin-low predictor to a publicly available database of histologically diverse subtypes of breast cancer (*n *= 113, NKI113) [14], which includes 10 medullary carcinomas, 20 metaplastic carcinomas and 22 invasive lobular carcinomas (ILC). Indeed, 8 (57%) of 14 and 2 (14%) of 14 claudin-low samples were identified as metaplastic and medullary carcinomas, respectively (Figure 4f and Table S5 in Additional file 1). Conversely, only 2 of a total of 22 ILCs were identified as claudin-low despite the lack of E-cadherin expression in lobular tumors [41].

### Claudin-low subtype resembles the MaSC profile

We hypothesized, as did Lim *et al. *[24], that a mammary differentiation program starting from a MaSC → luminal progenitor (pL) → mature luminal cells (mLs) exists, and therefore we built a differentiation model using data from Lim *et al. *[24]. For this predictor, higher scores represent greater differentiation status along this axis that culminates in ER+ mLs (Figure 4a). First, we applied this predictor onto similar subpopulations of purified human mammary epithelial cells that were separated by fluorescence-activated cell sorting (FACS) and profiled as part of the independent study of Raouf *et al. *[23] using different surface makers (Figure S7 in Additional file 1). The differentiation predictor showed 100% (8 of 8) accuracy with the bipotent progenitor subpopulation from Raouf *et al. *[23] [CD49f^+^(MUC1/CD133)^-^(CD10/THY1)^+^] showing the lowest differentiation score (and thus most similar to the MaSC fraction of Lim *et al. *[24]), followed by the luminal-restricted progenitor [CD49f^+^(MUC1/CD133)^+^(CD10/THY1)^-^] and then the mLs [CD49f^-^(MUC1/CD133)^+^(CD10/THY1)^-^]. In addition, we established and expression-profiled mammosphere cultures obtained from 14 different normal breast tissues because these cultures enrich for cells with stem or self-renewal capacity [15]. As expected, mammospheres showed a low differentiation score close to the MaSC profile (Figure 4b).

Notably, and as shown by Lim *et al. *[24], the breast cancer subtypes segregate along the normal mammary epithelial differentiation hierarchy starting with undifferentiated claudin-low tumors, followed by basal-like, then HER2-enriched tumors, and finally both luminal tumor subtypes (Figure 4c). As expected, we observed the same pattern using the breast cancer cell line data [21] (Figure 4d). Conversely, the nine-cell line claudin-low predictor identified the MaSC and stromal (CD49f^low^/EpCAM^-^) subpopulations of Lim *et al. *[24] as claudin-low, and as expected, both subpopulations showed the highest and lowest expression of the up- and downregulated genes that define the nine-cell line claudin-low predictor (Figure S8 in Additional file 1). Moreover, we applied the differentiation predictor to the mouse data set of Herschkowitz *et al. *[5] (Figure 4e) and observed that the previously identified claudin-low murine samples scored the lowest, while the MMTV-Neu and MMTV-PyMT models, which are known luminal mammary adenocarcinoma models, scored the highest. In addition, among 113 histological special types of breast cancer [14], medullary, adenoid cystic and metaplastic tumors showed the lowest score in the differentiation axis (Figure 4f), which is consistent with our previous reports of commonalities between metaplastic carcinomas, claudin-low tumors and breast cancer TICs [6].

**Figure 5:**
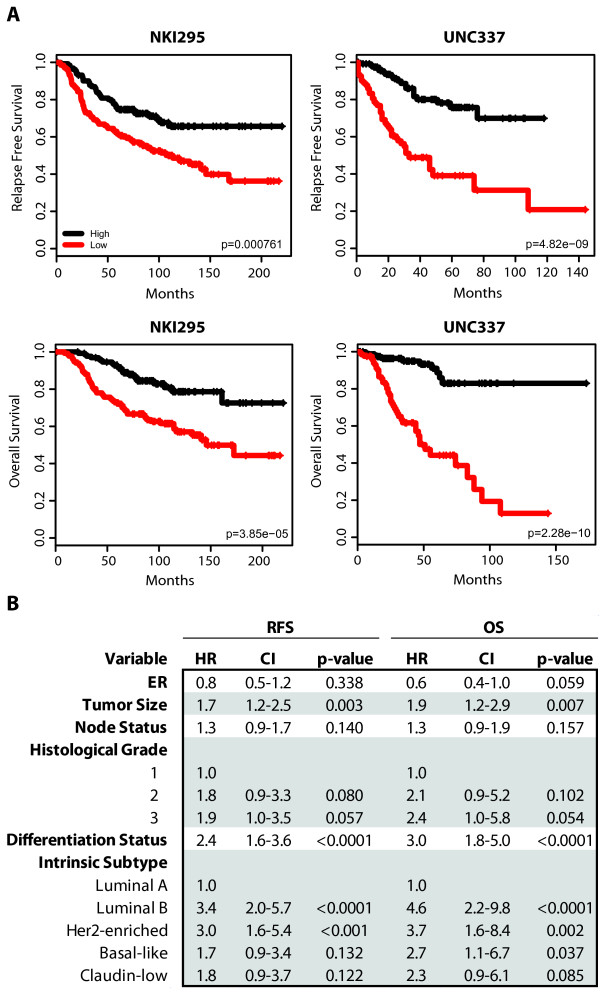
**RFS and OS of breast cancer patients based on the differentiation tumor status**. **(a) **Kaplan-Meier RFS and OS curves for UNC337 and NKI295 cohorts. Patients were rank-ordered and divided into two equal groups (low scores/differentiation in red and high scores/differentiation in black). **(b) **A combined multivariate analysis stratified by cohort was performed to test for significance of the differentiation status (as a continuous variable) conditioned on tumor intrinsic subtype, tumor size, histological grade, node status and ER. HR, hazard ratio; CI, confidence interval.

Finally, we evaluated the prognostic ability of the differentiation predictor in the UNC337 and NKI295 breast cancer patient data sets. Low differentiation scores were statistically significantly associated with poorer RFS and OS in univariate (Figure 5a) and multivariate analyses after adjusting for the main clinical-pathological parameters (i.e., size, grade, node and ER status), including tumor subtype (Figure 5b). These data suggest that tumors with an undifferentiated phenotype similar to the normal MaSC and/or early progenitors have a poorer prognosis compared to tumors with a more mature luminal phenotype, and this association is independent of the luminal B and HER2-enriched subtypes and the common clinical variables.

### Claudin-low and basal-like tumors are enriched with undifferentiated or mesenchymal cells

**Figure 6:**
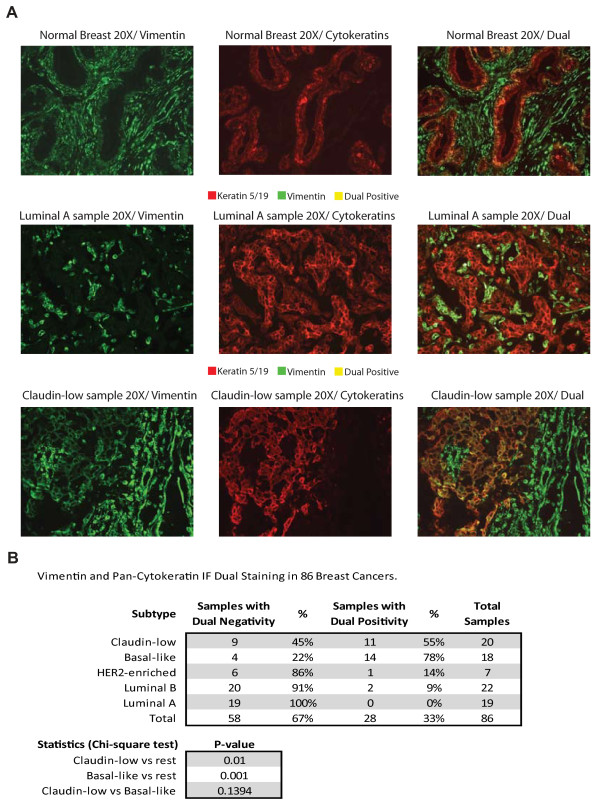
**Keratin 5/19 (red) and vimentin (green) immunofluorescence (IF) staining of 86 breast tumors, including 20 claudin-low tumor samples identified using the nine-cell line claudin-low predictor**. **(a) **Microscopic picture examples of individual and dual IF staining in one claudin-low sample with dual positive cells, and luminal A and normal breast samples without dual positive cells. **(b) **Tables summarizing the percentages of samples with negative and positive dual staining and the statistics.

Next, we sought to determine whether tumor cells with undifferentiated or mesenchymal features (as defined by immunofluorescence) exist within the different breast cancer intrinsic subtypes, similar to that performed by Creighton *et al. *[7]. Eighty-six breast tumors and one normal breast sample from the UNC337 database, including 20 claudin-low tumors, were evaluated using dual immunofluorescence (IF) staining with epithelial (keratin 5/19) and mesenchymal (vimentin) markers. Staining of the normal breast sample revealed that the antibody to vimentin stains the mesenchyme or stroma, whereas the antibody to keratin 5/19 stains the ducts, and no dual positive cells were seen (Figure 6). Conversely, 33% (28 of 86) of all tumor samples showed some degree of dual positivity, but 89% (25 of 28) of all samples with dual immunofluorescence positivity were either claudin-low (*n *= 11) or basal-like (*n *= 14). The remaining dual positive samples were identified in the HER2-enriched (*n *= 1) and luminal A subtypes (*n *= 2). Claudin-low tumors showed higher percentages of dual positive tumors than the other tumor subtypes when these are considered as a group (55% vs. 26%; *P *= 0.014, χ^2 ^test); however, no statistically significant differences in dual positivity were observed between claudin-low and basal-like tumors (55% vs. 78%; *P *= 0.14, χ^2 ^test). These data show that some epithelial tumor cells express mesenchymal features, these features are not due to contamination by adjacent stromal cells and almost all tumors with these features are basal-like or claudin-low tumors.

**Figure 7:**
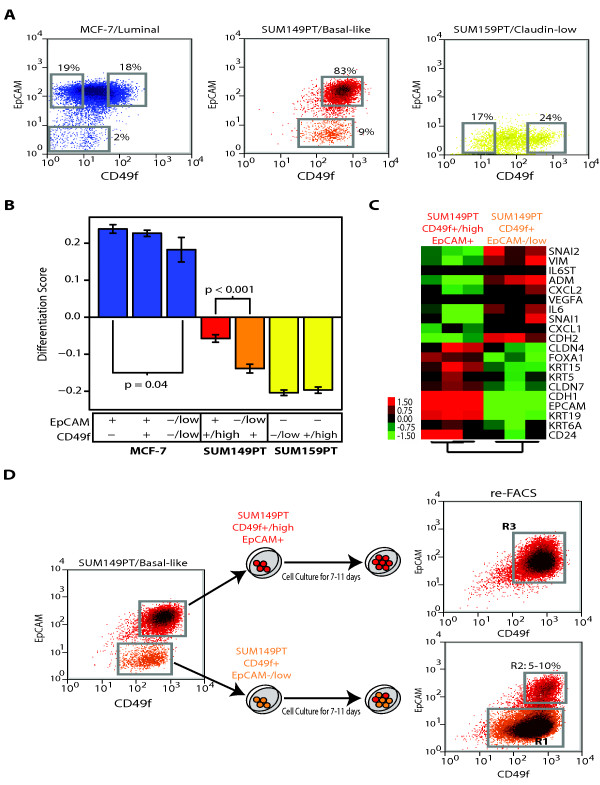
**FACS of breast cancer cell lines and characterization of their differentiation status**. **(a) **Expression of EpCAM and CD49f in MCF-7 (luminal), SUM149PT (basal-like) and SUM159PT (claudin-low) cell lines. The gates shown in each cell line (gray squares) represent the different sorted subpopulations that were further evaluated. **(b) **Differentiation scores of the different cell sorted subpopulations. Means and SD are shown for each subpopulation. Only significant *P *values (*P *< 0.05) are shown. **(c) **Gene expression analyses of the two FACS-sorted subpopulations within SUM149PT. A paired two-class SAM (FDR < 5%) was performed between both subpopulations in three independent experiments. **(d) ***In vitro *differentiation of CD49f^+^/EpCAM^-/low ^SUM149PT cells. The two SUM149PT sorted cell subpopulations were grown *in vitro *under the same conditions as before FACS. After 7-11 days in culture, expression of CD49 and EpCAM was reanalyzed in both subpopulations using FACS. Blue, MCF-7-sorted cell fractions; red, SUM149PT CD49f^+/high^EpCAM^+^-sorted subpopulation; orange, SUM149PT CD49f^+^/EpCAM^-/low^-sorted subpopulation; yellow, SUM159PT-sorted cell fractions. Similar results were obtained with and without supplemental FBS in the SUM149PT cell line.

We attempted to identify undifferentiated/mesenchymal epithelial cells within breast cancer cell lines. First, we analyzed the expression of surface markers CD49f and EpCAM (chosen based upon the studies of Lim *et al. *[24]) in three different cell lines: MCF-7 (luminal), SUM149PT (basal-like) and SUM159PT (claudin-low) (Figure 7). As expected on the basis of our previous genomic analysis of the differentiation status of these cell lines, virtually all claudin-low SUM159PT cells showed a stromal or MaSC antigenic phenotype (CD49f^+^/EpCAM^-^), ~98% of MCF-7 cells showed a mL phenotype (CD49f^-/low^/EpCAM^+^), and ~83% of SUM149PT cells showed a pL phenotype (CD49f^+^/EpCAM^+/high^). About 10% and ~2% of cells from SUM149PT and MCF-7 cell lines, respectively, showed low expression of EpCAM, suggesting that some cells within basal-like and luminal cell lines might have a more undifferentiated state. However, a clear EpCAM^-/low ^subpopulation was identified only in the SUM149PT cell line.

To further determine the differentiation status of the various cell subpopulations within MCF-7 (CD49f^-^/EpCAM^+^, CD49f^+^/EpCAM^+^, and CD49f^-/low^EpCAM^-/low^), SUM149PT (CD49f^+/high^/EpCAM^+ ^and CD49f^+^/EpCAM^-/low^) and SUM159PT (CD49f^-/low^/EpCAM^- ^and CD49f^+/high^/EpCAM^-^), we sorted and profiled these seven subpopulations using gene expression microarrays. The EpCAM^-/low ^cells derived from MCF-7 or SUM149PT lines showed a statistically significant undifferentiated state when compared with their EpCAM^+/high ^cell counterparts (Figure 7b); however, for MCF-7 cells, the EpCAM^-/low ^cells still showed high differentiation scores. Conversely, the CD49f^+^/EpCAM^-/low ^cells from the basal-like SUM149PT cell line showed the presence of a mesenchymal/claudin-low-like gene expression profile, with high expression of genes involved in wound response (i.e., interleukin 6, chemokine [C-X-C motif], ligand 1), angiogenesis (i.e., VEGFA) and extracellular matrix (i.e., vimentin, SNAI1), while genes involved in luminal differentiation (i.e., keratin 19, CD24) and cell-cell adhesion such as E-cadherin or claudin 7 were low (Figure 7c and Supplemental Data in Additional file 2). Since both claudin-low (CD49f^+^/EpCAM^-/low^) and basal-like (CD49f^+/high^/EpCAM^+^) cells exist within the SUM149PT cell line, we wished to determine whether one cell type gave rise to the other. When sorted and plated separately, 5 to 10% of the CD49f^+^/EpCAM^-/low ^SUM149PT cells differentiated into CD49f^+/high^/EpCAM^+ ^basal-like cells, whereas the CD49f^+/high^/EpCAM^+ ^basal-like cells maintained their differentiated status during *in vitro *culture (Figure 7d).

## Discussion

Here, claudin-low tumors were comprehensively characterized, and many important biological and clinical features were identified. Specifically, we addressed four topics for claudin-low tumors including (1) molecular features, (2) clinical and histological characteristics, (3) relation to established breast cancer cell lines and genetically engineered mouse models and (4) differentiation status based on analyses of purified normal mammary epithelial cell subpopulations.

Molecular characterization of the claudin-low subtype reveals that these tumors are significantly enriched in EMT and stem cell-like features while showing a low expression of luminal and proliferation-associated genes. Among these molecular characteristics, EMT and stem cell features have recently been linked to one another [18,33,42,43]. Indeed, expression of EMT-inducing transcription factors SNAI1 [33] or TWIST1 [33] or repression of E-cadherin [43] in mammary epithelial cells increases the number of stem cells, and these and other EMT-inducing transcription factors such as ZEB2 and TWIST2, as well as the mesenchymal marker vimentin, are expressed at higher levels in CD44^+^CD24^-/low ^stem cell-like cells than in more differentiated epithelial CD44^-^CD24^+ ^cells [18,33]. Consistent with this finding, we observed a high mRNA expression of known transcriptional repressors of E-cadherin such as SNAI1, SNAI2, TWIST1, TWIST2, ZEB1 and ZEB2, and other EMT-inducing factors such as hypoxia-inducible factor-1a in claudin-low tumors [31] (Figure 1b, Figure S2 in Additional file 1). Thus, our data suggest that claudin-low tumors, compared with the other intrinsic breast tumor subtypes, are the most enriched for stem cell and/or TIC features, and on the basis of our vimentin immunofluorescence staining, it appears that these mesenchymal features are present within epithelial cells, which is a feature not seen in normal breast tissues.

Acquisition of EMT and/or stem cell-like biological processes has been associated with therapeutic resistance [7,43,44]. We observed that claudin-low tumors do show a lower pCR rate than basal-like tumors (Figure 3a); however, the pCR rate of claudin-low tumors was roughly equivalent to that of the HER2-enriched subtype (without anti-HER2 therapies) and much higher than luminal A or luminal B tumors. Thus, as has been described for basal-like tumors [4], claudin-low tumors show some chemotherapy sensitivity, yet patients with these tumors still have poor survival outcomes overall (Figure 3b). A potential explanation for this similar scenario of basal-like and claudin-low tumors is that chemoresistant cells with TIC or mesenchymal properties are present at diagnosis in these two tumor subtypes as suggested by our immunofluorescence dual staining (Figure S9 in Additional file 1). This is also in concordance with a previous immunohistochemical study of 491 breast tumors where high expression of mesenchymal markers (i.e., vimentin, N-cadherin) and low expression of CDH1 were found almost exclusively in the triple-negative subgroup of tumors [45]. However, our treatment response data suggest that these tumor cells with mesenchymal properties within basal-like and claudin-low subtypes might not have the same treatment sensitivity to anthracycline/taxane-based chemotherapy. Thus, further studies are needed to better characterize the treatment sensitivity of claudin-low and basal-like tumors to specific chemotherapeutics and/or targeted therapies. The claudin-low nine-cell line centroid predictor developed here will assist immediately in identifying the claudin-low subtype and its possible predictive value in any neoadjuvant clinical trial with associated microarray data. However, we acknowledge a potential caveat of the nine-cell line claudin-low predictor, which is that tumors with high stromal content might also be identified as claudin-low. It is possible that the signature set of genes that are high in claudin-low tumors (and cell lines) are also high in nonepithelial cells, including fibroblasts and other mesenchyme-derived cells. Thus, we cannot rule out the possibility that some of the claudin-low tumors identified in this study are tumors with low epithelial and high myofibroblast content. It is also possible that this signature is one that can occur within epithelial cells, within stromal cells, or both. Special attention to the percentage of tumor cellularity of the sample being analyzed and/or strategies that can differentiate tumor cells with mesenchymal properties (i.e., immunoflourescence assays) from normal or tumor-associated fibroblasts with mesenchymal properties are needed for the further evaluation of this signature. Finally, from a translational point of view, it is interesting to note that the publicly available NCI-60 *in vitro *drug-screening database includes six breast cancer cell lines, four of which are claudin-low (BT549, MDA-MB-231, MDA-MB-435 and Hs578T) and two of which are luminal (MCF-7 and T47D). Among them, MDA-MB-435 cells have been shown to have melanoma characteristics [46], which is still a controversial topic [47]. Nonetheless, there is a need to develop better screening programs of drug sensitivity in breast cancer cell lines that resemble the basal-like subtype, as this subtype is missing from the NCI-60 set.

Invasive ductal, metaplastic and medullary or medullary-like claudin-low carcinomas share important biological relationships as defined by gene expression, suggesting that yet to be discovered common oncogenic changes might exist. Metaplastic and medullary carcinomas both have a high incidence of methylation of BRCA1 [48,49], and ~50% of breast tumors from BRCA1 mutation carriers show medullary-like features [50]. In addition, MDA-MB-436 and SUM1315MO2 claudin-low cell lines have mutations in BRCA1 [51]. Moreover, we have shown that BRCA1 mutant basal-like SUM149PT cell line has a small subpopulation of cells with mesenchymal/claudin-low-like features, and that these cells give rise to the basal-like cells that dominate these cultures. These data suggest that BRCA1 deficiency, which has been implicated in the differentiation of MaSC or bipotent progenitors into ER-positive luminal cells [52], might also contribute to the development or progression of undifferentiated claudin-low tumors and cell lines.

Although we have not performed functional tumor cell repopulating assays on human claudin-low tumors to show their enrichment for TICs because of the low incidence of these tumors (i.e., ~7 to 14%), there is, however, evidence that the claudin-low cell lines identified here show stem cell properties and may be highly enriched for TICs. For example, Charafe-Jauffret *et al. *[53] reported that in addition to having EMT features and high expression of stem cell markers such as ALDH1, many of these cell lines contain functional TICs. This is in concordance with another report [54] that showed that MDA-MB-231, SUM159PT and SUM1315MO2 have a high proportion (>90%) of CD44^+^/CD24^-/low ^cells, and that the CD44^+^/CD24^-/low ^subpopulation obtained from these cell lines was capable of self-renewal, forming tumors in nonobese diabetic severe combined immunodeficient mice, and were more resistant to chemotherapy.

Lim *et al. *[24] delineated a human mammary epithelial hierarchy by performing cell sorting on the basis of two cell surface markers (CD49f and EpCAM) and a series of *in vitro *and *in vivo *experiments, including gene expression profiling of different subpopulations of the normal breast. Using their microarray data, we developed a genomic differentiation predictor that classifies breast tumors on the basis of their differentiation status along a continuous MaSC → pL → mL epithelial hierarchy. We observed that the information provided by the differentiation status adds prognostic value even when considered with intrinsic subtype and the classical clinical variables. However, as developmental studies further characterize the normal mammary differentiation hierarchy, approaches such as the one reported here can be improved. For example, much less is known about other cell types in the normal breast, such as the myoepithelial progenitors and other potential intermediate progenitors, which may be responsible for the development of other rare breast cancer subtypes such as medullary carcinomas. Finally, a similar genomic approach based on FACS data coming from other developmental studies such as the ones by Lim *et al. *[24] or Raouf *et al. *[23] might prove useful in leukemia [55] or other solid tumors [56], where similar differentiation hierarchies have been identified, and thus this differentiation predictor algorithm may show benefit in cancers other than breast cancer.

Integration of the claudin-low tumor subtype together with the known intrinsic subtypes delineates a differentiation hierarchy that resembles the normal epithelial development. These data point to different cells of origin for each intrinsic subtype, or different stages of developmental arrest for each subtype with a common cell type of transformation, or some combination of the two as different processes may be occurring for each different subtype. Indeed, Lim *et al. *[24] suggested that the potential cell of origin of the basal-like subtype in BRCA1 carriers might be the pL instead of the MaSC. Alternatively, as suggested by our *in vitro *analyses of the SUM149PT cell line, BRCA1-mutated basal-like tumors might arise from transformation of a MaSC that is similar to claudin-low tumors or cell lines, but the claudin-low tumors stay arrested in this undifferentiated state, while MaSC or claudin-low cells in basal-like tumors are able to divide asymmetrically and give off differentiated progeny that then arrest at the pL state [57]. The therapeutic implication of the claudin-low subtype will require additional retrospective and prospective evaluations, but what does appear clearer is that the intrinsic subtypes of breast cancer may be reflective of distinct stages of mammary epithelial cell development and that the claudin-low tumors (and cell lines) show the least differentiated phenotype.

## Conclusions

It has become appreciated that breast cancer is not one disease, but in fact represents multiple disease types, each of which may require a unique treatment. In this article, we characterize an important new disease group, namely the claudin-low subtype of breast cancer, and show that these tumors have a poor prognosis and features of mesenchymal and mammary stem cells. We also provide new tools for the identification and study of this subtype in tumors and cell lines.

## Abbreviations

BL: basal-like; CDH1: E-cadherin; CL: claudin-low; CLDN3: claudin 3; CLDN4: claudin 4; CLDN7: claudin 7; DWD: distance-weighted discrimination; EMT: epithelial-to-mesenchymal transition; EpCAM: epithelial cell adhesion molecule; ER: estrogen receptor; FACS: fluorescence-activated cell sorting; FDR: false discovery rate; GEO: Gene Expression Omnibus; H2: HER2-enriched; HER2: epidermal growth factor receptor 2; HR: hazard ratio; IF: immunofluorescence; ILC: invasive lobular carcinoma; IRB: Institutional Review Board; KRT14: keratin 14; KRT17: keratin 17; KRT18: keratin 18; KRT19: keratin 19; KRT5: keratin 5; LA: luminal A; LB: luminal B; MaSC: mammary stem cell; mL: mature luminal cell; mRNA: messenger RNA; NBL: normal breast-like; pCR: pathological complete response; pL: luminal progenitor; PR: progesterone receptor; RMA: robust multiarray analysis; SAM: significance analyses microarrays; SNAI1: Snail 1; SNAI2: Snail 2; TIC: tumor-initiating cell; UNC: University of North Carolina; VIM: vimentin.

## Competing interests

CMP is a major stockholder of BioClassifier LLC and co-founder and managing partner of University Genomics. CMP and JSP have filed a patent on the PAM50 assay (University of North Carolina) and on intrinsic subtyping (University of Utah).

## Authors' contributions

AP, JSP, OK and CMP contributed to experimental design. AP, JSP, OK, CL, JIH and XH were responsible for performing experiments. AP, JSP, OK and CF contributed to data analysis. AP and CMP contributed to manuscript preparation.

## Supplementary Material

Additional file 1 **Supplementary Tables S1-S5 and Supplementary Figures S1-S10**. Table S1. Biological processes and signaling pathways enriched in claudin-low vs. basal-like tumors. Table S2. Biological processes and signaling pathways enriched in claudin-low tumors vs. rest. Table S3. Identification of the claudin-low subtype in a panel of breast cancer cell lines. Table S4. Histological examination of claudin-low tumors. Table S5. Evaluation of the intrinsic breast cancer molecular subtypes in histologically diverse types. Figure S1. Intrinsic unsupervised hierarchical clustering of the UNC337 database. Figure S2. Average expression of additional selected genes and gene signatures across the breast cancer subtypes. Figure S3. E-cadherin and claudin 3 immunohistochemical staining of breast tumors. Figure S4. Intrinsic gene set analysis of 52 breast cancer cell lines. Figure S5. Claudin-low tumor and normal breast predictions in 52 breast cancer cell lines. Figure S6. Average expression of genes and gene signatures across the various mouse classes. Figure S7. Differentiation predictions in Raouf *et al. *[23] database. Figure S8. Expression of the nine-cell line claudin-low predictor across different subpopulations of the normal breast. Figure S9. Mean expression of the top highly expressed (*n *= 833) and low expressed (*n *= 642) genes in claudin-low cell lines across 337 human breast tumor samples classified according to intrinsic subtype, including the normal breast-like group. Figure S10. Localization of five claudin-low samples (BC00054, 020018B, BC00075, 010384B, and BC00083) in the UNC337 intrinsic clustering. Click here for file

Additional file 2 **Supplemental Data**. Clinical data and gene lists reported throughout the manuscript. Click here for file
