# Supplementary material for: Phenotypic and molecular characterization of the claudin-low intrinsic subtype of breast cancer
Source: Breast Cancer Res. 2010 Sep 2;12(5):R68. doi: 10.1186/bcr2635 (PMC3096954; doi:10.1186/bcr2635)

Supplementary Material for:

Phenotypic and Molecular Characterization of the Claudin-low Intrinsic Subtype of Breast Cancer

Aleix Prat, Joel S. Parker, Olga Karginova, Cheng Fan, Chad Livasy, Jason I. Herschkowitz, Xiaping He and Charles M. Perou*

*To whom correspondence should be addressed. E-mail: cperou@med.unc.edu

This PDF file includes:

- Table S1. Biological processes and signaling pathways enriched in Claudin-low vs. Basal-like tumors.
- Table S2. Biological processes and signaling pathways enriched in Claudin-low tumors vs. rest.
- Table S3. Identification of the Claudin-low subtype in a panel of breast cancer cell lines.
- Table S4. Histological examination of Claudin-low tumors.
- Table S5. Evaluation of the intrinsic breast cancer molecular subtypes in histologically diverse types.

- Figure Legends
- Fig. S1. Intrinsic unsupervised hierarchical clustering of the UNC337 database.
- Fig. S2. Average expression of additional selected genes and gene signatures across the breast cancer subtypes.
- Fig. S3. E-Cadherin and Claudin 3 immunohistochemical staining of breast tumors.
- Fig. S4. Intrinsic gene set analysis of 52 breast cancer cell lines.
- Fig. S5. Claudin-low tumor and Normal Breast predictions in 52 breast cancer cell lines.
- Fig. S6. Average expression of genes and gene signatures across the various mouse classes.
- Fig. S7. Differentiation predictions in Raouf et al. database.
- Fig. S8. Expression of the 9-Cell Line Claudin-low predictor across different subpopulations of the normal breast.
- Fig. S9. Mean expression of the top highly expressed and low expressed genes in Claudin-low cell lines across 337 human breast tumor samples.
- Fig. S10. Localization of five H/E Claudin-low samples in the UNC337 intrinsic clustering.
- References

Table S1. Biological Processes (BP) and Signaling Pathways Enriched in Claudin-low vs Basal-like Tumors*

Upregulated (1,190 genes)

GO BP Terms	Count	EASE score	KEGG Pathway Terms	Count	EASE score
Response to wounding	96	3.14E-28	Hematopoietic cell lineage	27	3.69E-08
Inflammatory response	70	9.14E-22	Cell adhesion molecules (CAMs)	32	6.71E-07
Cell communication	363	2.84E-14	Natural killer cell mediated cytotoxicity	27	1.64E-04
Developmental process	299	1.79E-13	Leukocyte transendothelial migration	25	2.21E-04
Cell adhesion	87	1.20E-07			
T cell differentiation	14	8.51E-06			
B cell mediated immunity	16	1.15E-06			

Downregulated (526 genes)

GO BP Terms	Count	EASE score	KEGG Pathway Terms	Count	EASE score
Cell cycle phase	55	4.70E-26	Cell Cycle	20	4.57E-10
Mitotic cell cycle	47	2.00E-21	p53 signaling pathway	9	7.95E-04
Spindle organization and biogenesis	11	2.12E-11	Tight junction	11	0.005

*Gene lists were selected after perfoming SAM (FDR 0%) between Claudin-low tumors as defined by SigClust versus Basal-like tumors. Selected Gene Ontology (GO) and Kyoto Encyclopedia of Genes and Genomes (KEGG) pathway terms with p-values of < 0.001 and <0.05, respectively, are shown here. The complete GO BP terms, genes and p-values are found in Supplemental Data.

Table S2. Biological Processes (BP) and Signaling Pathways Enriched in Claudin-low Tumors*

Upregulated (1,308 genes)

GO BP Terms	Count	EASE score	KEGG Pathway Terms	Count	EASE score
Immune system process	220	8.92E-46	Hematopoietic cell lineage	39	2.82E-15
Inflammatory response	93	7.52E-36	Cytokine-cytokine receptor interaction	63	9.66E-10
Cell communication	450	1.41E-29	Cell adhesion molecules (CAMs)	40	1.71E-09
Lymphocyte activation	53	6.05E-19	Natural killer cell mediated cytotoxicity	39	8.03E-09
Developmental process	332	4.21E-15	Leukocyte transendothelial migration	33	8.31E-07
Cell differentiation	210	5.22E-14	Antigen processing and presentation	21	3.76E-04
Cell adhesion	109	1.16E-12	T cell receptor signaling pathway	24	1.96E-04
T cell differentiation	21	3.53E-11			
Vasculature development	40	7.78E-10			
Cell death	105	2.29E-09			
B cell mediated immunity	19	2.02E-08			

Downregulated (359 genes)

GO BP Terms	Count	EASE score	KEGG Pathway Terms	Count	EASE score
Chromatin assembly	11	7.70E-06	Tight junction	9	0.003
Nucleosome assembly	10	1.72E-05	Cell adhesion molecules (CAMs)	8	0.01
Protein-DNA complex assembly	11	2.60E-04	Adherens junction	6	0.01
Cell adhesion	28	2.34E-04			

*Gene lists were selected after perfoming SAM (FDR 0%) between Claudin-low tumors as defined by SigClust versus the rest. Selected Gene Ontology (GO) and Kyoto Encyclopedia of Genes and Genomes (KEGG) pathway terms with p-values of < 0.001 and <0.05, respectively, are shown here. The complete GO BP terms, genes and p-values are found in Supplemental Data.

Table S3. Identification of the Claudin-low Subtype in a Panel of Breast Cancer Cell Lines

Cell Lines	(Neve et al., 2006)*[1]	(Sieuwerts et al., 2009)*[2]	CD44/CD24 mRNA Ratio*[3]	CD49f/EpCAM mRNA Ratio*[3]	Intrinsic Subtype*[4]
MDA-MB-435s	Basal B	Normal-like	1.84	1.77	Claudin-low
MDA-MB-436	Basal B	Normal-like	1.11	1.28	Claudin-low
Hs578T	Basal B	Normal-like	1.58	1.34	Claudin-low
BT549	Basal B	Normal-like	1.28	1.59	Claudin-low
MDA-MB-157	Basal B	Normal-like	1.28	1.51	Claudin-low
SUM1315MO2	Basal B	Normal-like	1.96	1.48	Claudin-low
MDA-MB-231	Basal B	Normal-like	1.37	1.17	Claudin-low
SUM159PT	Basal B	Normal-like	1.51	1.47	Claudin-low
HBL100	Basal B	-	0.96	1.38	Claudin-low
HCC1500	Basal B	-	1.27	0.89	-
HCC38	Basal B	-	0.8	0.66	-
SUM149PT	Basal B	Basal-like	1.04	0.66	-
MDA-MB-468	Basal A	Basal-like	0.83	0.66	-
BT20	Basal A	Basal-like	0.84	0.71	-
HCC1937	Basal A	Basal-like	0.72	0.76	-
HCC1187	Basal A	-	0.53	0.51	-
HCC1569	Basal A	-	1.02	0.67	-
HCC2157	Basal A	-	0.71	0.68	-
HCC3153	Basal A	-	0.71	0.63	-
HCC70	Basal A	-	0.75	0.62	-
HCC1008	-*[5]	-	0.62	0.66	-
DU4475	-*[5]	-	0.62	0.55	-
HCC1599	-*[5]	-	0.66	0.49	-
HCC1954	Basal A	-	1.03	0.77	-
SUM190PT	Basal A	-	0.84	0.60	-
HCC1143	Basal A	-	0.64	0.60	-
SUM225CWN	Basal A	-	0.64	0.71	-
MPE600	Luminal	Luminal	0.53	0.44	-
CAMA-1	Luminal	Luminal	0.5	0.48	-
ZR75-1	Luminal	Luminal	0.62	0.48	-
MDA-MB-361	Luminal	Luminal	0.55	0.54	-
MDA-MB 175 VII	Luminal	Luminal	0.47	0.48	-
MDA-MB-415	Luminal	Luminal	0.61	0.62	-
SUM185PE	Luminal	Luminal	0.47	0.42	-
BT474	Luminal	Luminal	0.48	0.55	-

MCF-7	Luminal	Luminal	0.58	0.45	-
HCC1007	Luminal	-	0.56	0.37	-
HCC1428	Luminal	-	0.7	0.48	-
HCC202	Luminal	-	0.54	0.50	-
LY2	Luminal	-	0.62	0.38	-
SUM44PE	Luminal	-	0.67	0.60	-
SUM52PE	Luminal	-	0.51	0.56	-
SK-BR-3	Luminal	Her2+	0.49	0.42	-
UACC812	Luminal	-	0.51	0.63	-
ZR7530	Luminal	-	0.62	0.49	-
ZR75B	Luminal	-	0.46	0.47	-
MDA-MB-134 VI	Luminal	Luminal	0.56	0.60	-
T47D	Luminal	Luminal	0.55	0.52	-
MDA-MB-453	Luminal	Her2+	0.41	0.64	-
AU565	Luminal	-	0.5	0.46	-
BT483	Luminal	-	0.5	0.49	-
HCC2185	Luminal	-	0.43	0.46	-
EVSA-T	-	Her2+			-
MDA-MB-330	-	Her2+			-
UACC893	-	Her2+			-
SK-BR-7	-	Normal-like			-
SK-BR-5	-	Luminal			-
OCUB-F	-	Luminal			-
SUM229PE	-	Basal-like			-

*[1], Subtype calls of the different breast cancer cell lines were identified in Neve et al. (*1*) by performing an unsupervised hierarchical clustering of 1,438 probes which showed substantial variation across the data (4 measurements that varied by more than Log2 1.89). *[2], Subtype calls of the different breast cancer cell lines were derived in Sieuwerts et al. (*2*) using the intrinsic list of Perou et al. (*3*). *[3], mRNA ratios were derived from the array data of Neve et al. (*1*). *[4], The Claudin-low subtype classification was based on unsupervised hierarchical clustering using the intrinsic list of Parker et al. (*4*) and the node identified in fig. S4. *[5], Breast cancer cell lines not included in Neve et al. (*1*).

Table S4. Histological Examination of Claudin-low Tumors in UNC337*

Sample	Histology Review	Tumor Border	Lymphoid Infiltration	Necrosis	ER Status	Node Status	Grade	Tumor size	PAM50 call
1	Ductal	I	P	P	0	1	3	2	HER2-enriched
2	Ductal	I	A	A	0	1	3	NA	NBL
3	Ductal	I	A	P	0	0	3	NA	NBL
4	Ductal	I	A	A	1	1	3	2	NBL
5	Ductal	I	A	A	1	1	2	4	NBL
6	Ductal	Pu	A	A	NA	NA	NA	NA	BL
7	Ductal	I	P	P	0	0	2	1	BL
8	Ductal	I	A	A	0	0	3	1	NBL
9	Ductal	I	A	A	1	1	3	4	NBL
10	Ductal	I	A	A	1	0	3	2	NBL
11	Ductal	I	A	P	0	0	3	4	NBL
12	Ductal in LN	NA	NA	A	NA	1	NA	1	LB
13	Ductal with MF	Pu	P	P	0	0	NA	1	NBL
14	Ductal with MF	Pu	P	P	0	1	3	2	HER2-enriched
15	Ductal with MF	Pu	P	A	0	0	3	1	BL
16	Ductal with MF	Pu	P	A	0	1	3	2	BL
17	Ductal with MF	Pu	P	A	0	0	3	2	BL
18	Metaplastic	Pu	A	P	0	0	3	2	BL
19	Metaplastic*[1]	NA	NA	NA	0	0	NA	2	BL
20	Metaplastic*[1]	NA	NA	NA	0	0	3	2	BL
21	Metaplastic*[1]	NA	NA	NA	0	0	3	4	NBL
22	Metaplastic*[1]	NA	NA	NA	0	0	3	2	BL
23	Metaplastic*[1]	NA	NA	NA	0	1	3	4	BL
24	Metaplastic*[1]	NA	NA	NA	0	0	NA	2	BL
25	Metaplastic*[1]	NA	NA	NA	0	0	3	2	BL
26	Metaplastic*[1]	NA	NA	NA	0	0	3	3	BL
27	Metaplastic in LN	NA	NA	P	NA	1	NA	1	NBL
28	Micropapillary	I	A	A	0	0	3	2	NBL
29	Mixed Ductal/Lobular	I	A	A	0	1	2	3	NBL

*Histological diagnoses are based on the WHO histological classification of tumors of the breast. Claudin-low tumors have been identified by the 9-Cell Line Claudin-low Predictor. LN, lymph node; MF, medullary features have been defined as the presence of pushing margins and brisk tumor lymphocytic infiltration without meeting the criteria for being classified as medullary carcinoma; Pu, pushing; I, infiltrative; P, present; A, absent; Tumor size (0, <2 cm; 1, 2-5 cm; 3, >5 cm), Node status (0, none; 1, ≥ 1 positive node), ER status (0, negative; 1, positive), Grade (1, low grade; 2, intermediate grade; 3, high grade); NA, not available; BL, Basal-like; NBL, Normal Breast-like; LB, Luminal B. *[1], tumors whose histological diagnosis was only available from clinical reports.

Table S5. Evaluation of the Intrinsic Breast Cancer Molecular Subtypes in Histologically Diverse Types.

	Claudin-low	Basal-like	HER2-enriched	Luminal B	Luminal A	Normal Breast-like
**Metaplastic**	8 (57%)	11 (46%)	-	-	-	1 (17%)
**Medullary**	2 (14%)	8 (33%)	-	-	-	-
**ILC**	2 (9%)	1 (4%)	5 (50%)	3 (17%)	8 (21%)	3 (50%)
**Tubular**	1 (7.1%)	-	-	-	7 (18%)	1 (17%)
**Adenocystic**	-	4 (17%)	-	-	-	-
**Apocrine**	-	-	3 (30%)	1 (6%)	2 (5%)	-
**Neuroendocrine**	-	-	-	2 (11%)	7 (18%)	1 (17%)
**IDC with OGC***	-	-	-	4 (22%)	1 (3%)	-
**Micropapillary**	-	-	2 (20%)	3 (17%)	3 (8%)	-
**Mucinous A**	1 (7.1%)	-	-	3 (17%)	6 (15%)	-
**Mucinous B**	-	-	-	2 (11%)	7 (18%)	-

*ILC, invasive lobular carcinoma; IDC with OGC, invasive ductal carcinoma with osteoclastic giant cells.

Figure Legends

Figure S1. Intrinsic unsupervised hierarchical clustering of the UNC337 database. Average-linkage hierarchical clustering of genes and arrays was performed using the intrinsic gene list from Parker et al. (*4*) on the 320 breast tumors and 17 normal breast tissue samples (UNC337). Claudin-low breast cancer intrinsic molecular subtype was defined by SigClust (*5*) (Yellow color array tree node, $P < 0.0001$). Approximate localization of characteristic gene clusters is shown on the right side of the figure. Gene symbols of important Claudin-low gene clusters are also shown. The Treeview files of this clustering can be obtained in the UMD UNC https://genome.unc.edu/ website.

Figure S2. Average expression of additional selected genes and gene signatures (*6-9*) across the intrinsic breast cancer subtypes including the Claudin-low group defined by SigClust (*5*) and the Normal Breast-like group. P-values shown here have been calculated by comparing gene expression means across all subtypes.

Figure S3. E-Cadherin (CDH1) and Claudin 3 (CLDN3) immunohistochemical staining of 103 breast tumors, including 22 Claudin-low samples identified by SigClust (*5*). (**A**) Light microscopic picture examples (20X) of negative/weak and moderate/strong positive staining for CDH1 and CLDN3 in two Claudin-low, one Luminal B and one Basal-like tumor samples. (**B**) Tables summarizing the IHC scores and the statistics.

Figure S4. Intrinsic gene set analysis of 52 breast cancer cell lines. (**A**) The intrinsic list of Parker et al. (*4*) was used to hierarchically cluster 52 breast cancer cell lines from Neve et al. (*1*) Average-linkage clustering was performed on genes and arrays. In the tree, the yellow node denotes the Claudin-low cell lines (cluster correlation ~59%). The Treeview files of this clustering can be obtained in the UMD UNC https://genome.unc.edu/ website. (**B**) Expression of selected genes associated with luminal differentiation (KRT8, KRT5, KRT14, KRT19, ESR1, ERBB2), EMT (CDH1, CLDN3, CLDN4, CLDN7, VIM, TWIST1, SNAI1, SNAI2, ZEB1, ZEB2) and stem cell and/or TICs features (CD44, CD24, ALDH1A1, EPCAM) across the cell line database. (**C**) Table summarizing the cell lines selected for building the 9-Cell Line Claudin-low predictor. Among them, MDA-MB-435 cells have been shown to have melanoma characteristics (*10*), which is still a controversial topic (*11*).

Figure S5. Claudin-low tumor and Normal Breast predictions in 52 breast cancer cell lines (*1*). In order to build the Claudin-low tumor predictor, we first selected those genes that were significantly differentially expressed between Claudin-low tumors defined by SigClust (or cell lines) and all other subtypes using a two-class, unpaired SAM, with <5% FDR. Then we used these gene lists and built two centroids for Claudin-low vs. “others” and used these as our training data. For every sample, we calculated the euclidean distances to the two centroids, and defined each sample as Claudin-low if its nearest centroid was the Claudin-low centroid ([Distance to the “others” centroid] / [Distance to the Claudin-low centroid] ≥ 1.0). Distance weighted discrimination (DWD; (https://genome.unc.edu/pubsup/dwd/) was used to calculate the distance to each centroid. Using the same methodology, we also build a normal breast predictor by selecting those genes that were significantly differentially expressed between normal breast tissues and breast tumors using a two-class, unpaired SAM, with 0% FDR; note that these gene lists are also included in Supplemental Data.

Figure S6. Average expression of important genes and gene signatures across the various mouse classes (I-X) previously published (*12*). (**A**) Classical markers used to characterize breast tumors are shown for mRNA expression levels for: basal markers (keratins 5 [KRT5], 14 [KRT14] and 17 [KRT17]), luminal markers (keratins 18 [KRT18] and 19 [KRT19]), estrogen receptor (ESR1), progesterone receptor (PR), GATA3 and HER2 (ERBB2); to the right is shown a box-and-whisker plot for expression of the previously published luminal and proliferation gene signatures. (**B**) Markers of epithelial-to-mesenchymal transition (Vimentin [VIM], Snail-1 [SNAI1], Snail-2 [SNAI2], TWIST2, ZEB1, ZEB2, E-Cadherin [CDH1], and Claudins 3 [CLDN3], 4 [CLDN4] and 7 [CLDN7]), and to the right, expression of stromal- and immune-related signatures (*7, 9, 13*). (**C**) Markers of stem cells / cancer stem cells / epithelial differentiation (CD44, EPCAM, CD10, CD49f, CD29, CD133, MUC1, THY1, and ALDH1A1), and expression to the right, published CSC signatures (*6, 8, 14*). Each colored square on the left side panels represents the relative transcript abundance (in log 2 space) with highest expression being red, average expression being black, and lowest expression being green. Group I, Murine Normal breast samples; Group II, Claudin-low samples; Group III, DMBA/Wnt1; Group IV, BRCA1/p53/Wnt1; Group V, p53null/p53het IR; Group VI, MMTV-Neu/PyMT; Group VII, WAP-Myc; Group VIII, WAP-Int3; Group IX, WAPT121/WAPTag; Group X, TgC3(1)-Tag. P-values (Student's t-test) shown here have been calculated by comparing gene expression means across all mouse classes. *, Statistical significant p-values (<0.05, Student's t-test) obtained by comparing Group II (Claudin-low) vs. Group I (Murine Normal breast).

Figure S7. Differentiation predictions in Raouf et al. (*15*) bipotent progenitor subpopulation, luminal restricted progenitor subpopulation and mature luminal cell subpopulation. Note: an outlier mL subpopulation identified in Raouf et al. (*15*) (Diff Luminal-1) has been removed from this analysis. mL, mature luminal; pL, luminal progenitor.

Figure S8. Expression of the 9-Cell Line Claudin-low predictor across different subpopulations of the normal breast. (**A**) Lim et al. (*16*) subpopulations. (**B**) Raouf et al. (*15*) subpopulations. MaSC, mammary stem cell; mL, mature luminal; pL, luminal progenitor; Str, stromal; mM, mature myoepithelial. Yellow color bars denote those subpopulations identified as Claudin-low by the 9-Cell Line Claudin-low predictor. Note: an outlier mL subpopulation identified in Raouf et al. (*15*) (Diff Luminal-1) has been removed from this analysis.

Figure S9. Mean expression of the top highly expressed (n=833) and low expressed (n=642) genes in Claudin-low cell lines across 337 human breast tumor samples classified according to intrinsic subtype, including the Normal Breast-like group. Both gene lists were obtained by performing Significance Analysis Microarray (SAM) between Claudin-low breast cancer cell lines vs. the rest (FDR<5%). BL, Basal-like; CL, Claudin-low; H2, HER2-enriched; LA, Luminal A; LB, Luminal B; NBL, Normal Breast-like

Figure S10. Localization of five Claudin-low samples (BC00054, 020018B, BC00075, 010384B, and BC00083) in the UNC337 intrinsic clustering. The H/E (haematoxylin and eosin) slides of these five samples have been previously reported in Herschkowitz et al., Genome Biol 2007, Supplemental File 7. Red: the five Claudin-low samples; Yellow, the Claudin-low intrinsic cluster as defined by SigClust.

Figure S1

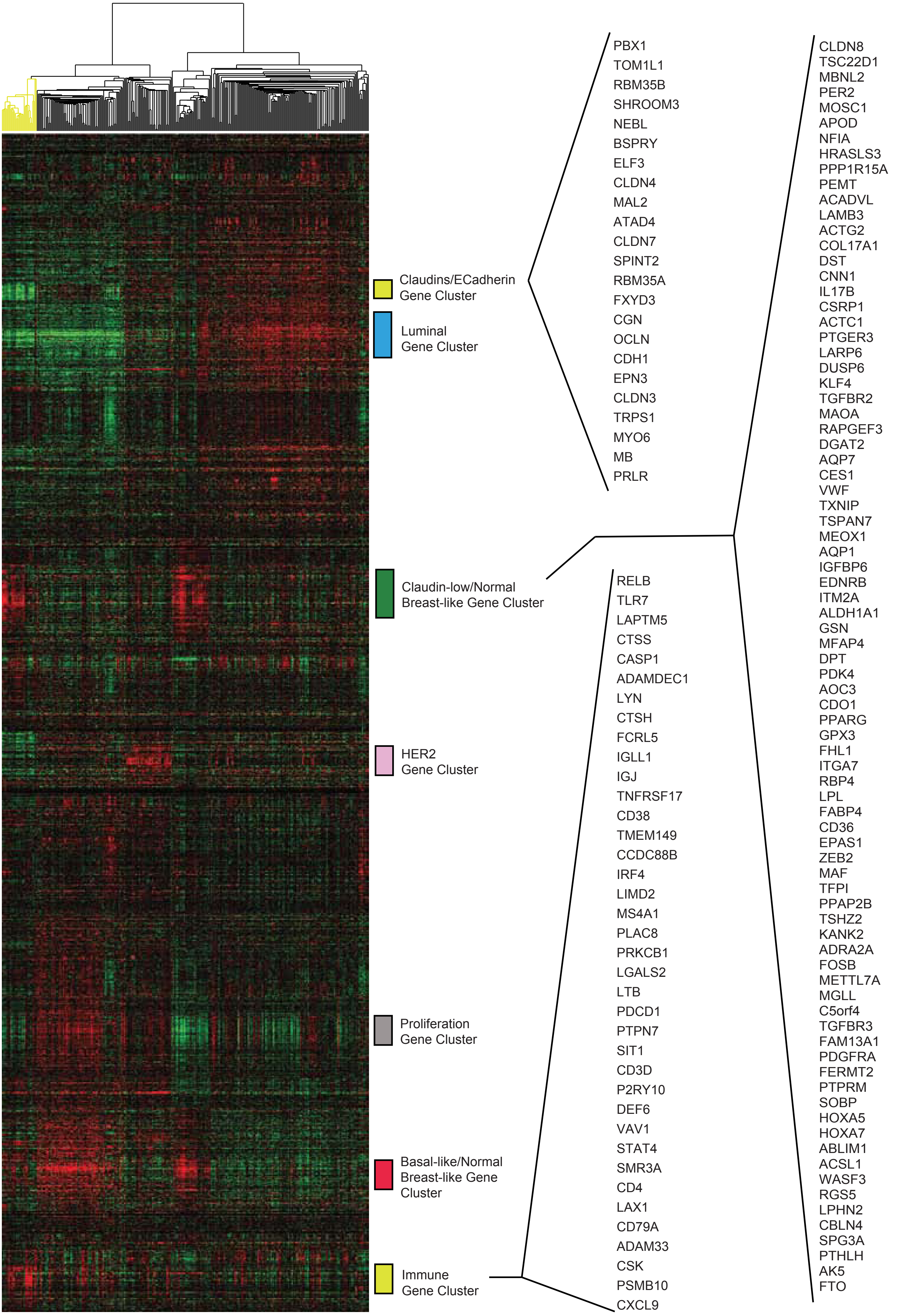

Claudins/ECadherin Gene Cluster
Luminal Gene Cluster
Claudin-low/Normal Breast-like Gene Cluster
HER2 Gene Cluster
Proliferation Gene Cluster
Basal-like/Normal Breast-like Gene Cluster
Immune Gene Cluster
PBX1
TOM1L1
RBM35B
SHROOM3
NEBL
BSPRY
ELF3
CLDN4
MAL2
ATAD4
CLDN7
SPINT2
RBM35A
FXYD3
CGN
OCLN
CDH1
EPN3
CLDN3
TRPS1
MYO6
MB
PRLR
RELB
TLR7
LAPTM5
CTSS
CASP1
ADAMDEC1
LYN
CTSH
FCRL5
IGLL1
IGJ
TNFRSF17
CD38
TMEM149
CCDC88B
IRF4
LIMD2
MS4A1
PLAC8
PRKCB1
LGALS2
LTB
PDCD1
PTPN7
SIT1
CD3D
P2RY10
DEF6
VAV1
STAT4
SMR3A
CD4
LAX1
CD79A
ADAM33
CSK
PSMB10
CXCL9
CLDN8
TSC22D1
MBNL2
PER2
MOSC1
APOD
NFIA
HRASLS3
PPP1R15A
PEMT
ACADVL
LAMB3
ACTG2
COL17A1
DST
CNN1
IL17B
CSRP1
ACTC1
PTGER3
LARP6
DUSP6
KLF4
TGFBR2
MAOA
RAPGEF3
DGAT2
AQP7
CES1
VWF
TXNIP
TSPAN7
MEOX1
AQP1
IGFBP6
EDNRB
ITM2A
ALDH1A1
GSN
MFAP4
DPT
PDK4
AOC3
CDO1
PPARG
GPX3
FHL1
ITGA7
RBP4
LPL
FABP4
CD36
EPAS1
ZEB2
MAF
TFPI
PPAP2B
TSHZ2
KANK2
ADRA2A
FOSB
METTL7A
MGLL
C5orf4
TGFBR3
FAM13A1
PDGFRA
FERMT2
PTPRM
SOBP
HOXA5
HOXA7
ABLIM1
ACSL1
WASF3
RGS5
LPHN2
CBLN4
SPG3A
PTHLH
AK5
FTO

Figure S2

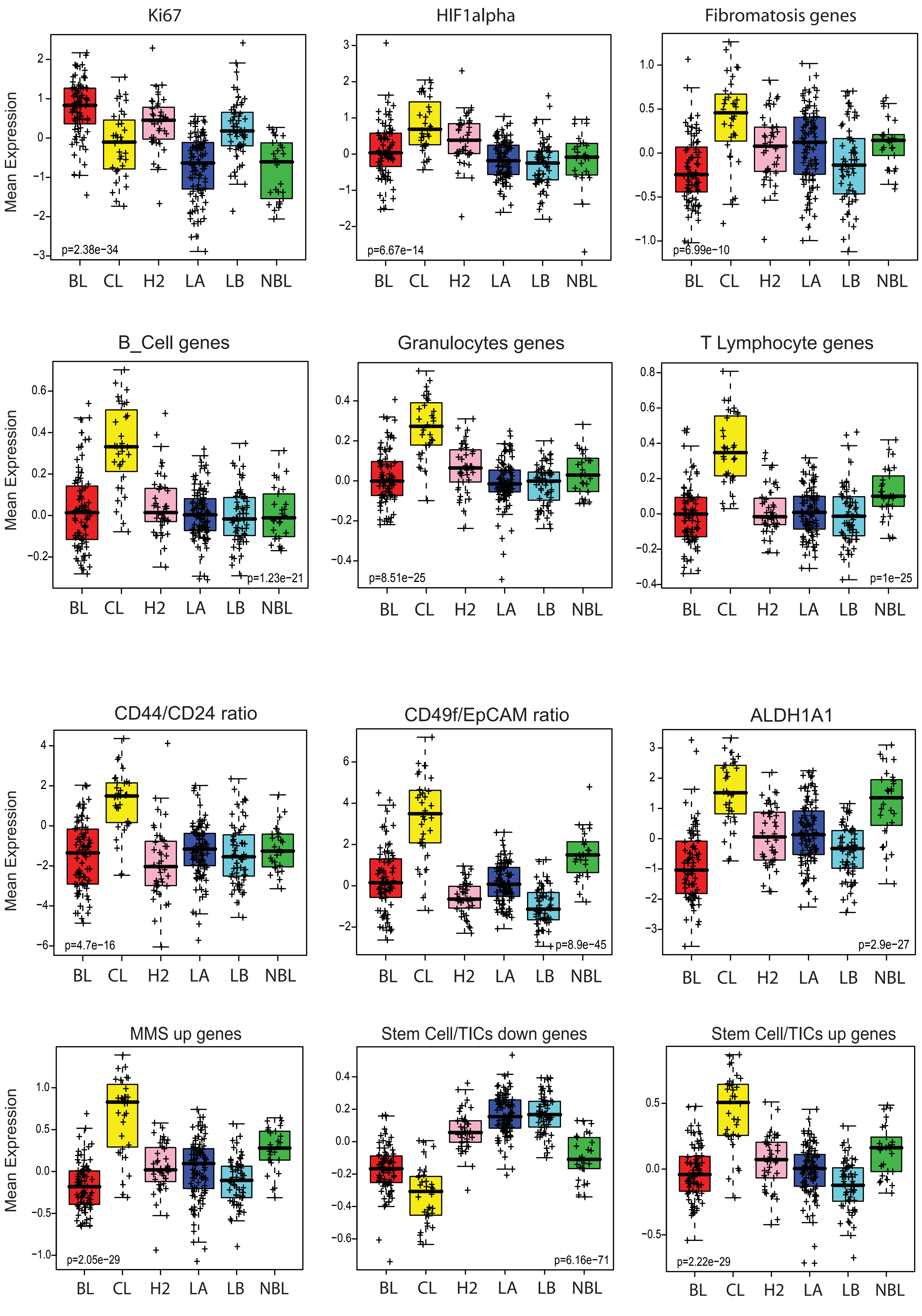
Ki67
HIF1alpha
Fibromatosis genes
B_Cell genes
Granulocytes genes
T Lymphocyte genes
CD44/CD24 ratio
CD49f/EpCAM ratio
ALDH1A1
MMS up genes
Stem Cell/TICs down genes
Stem Cell/TICs up genes
Mean Expression
BL CL H2 LA LB NBL
p=2.38e−34
p=6.67e−14
p=6.99e−10
p=1.23e−21
p=8.51e−25
p=1e−25
p=4.7e−16
p=8.9e−45
p=2.9e−27
p=2.05e−29
p=6.16e−71
p=2.22e−29

Figure S3

A

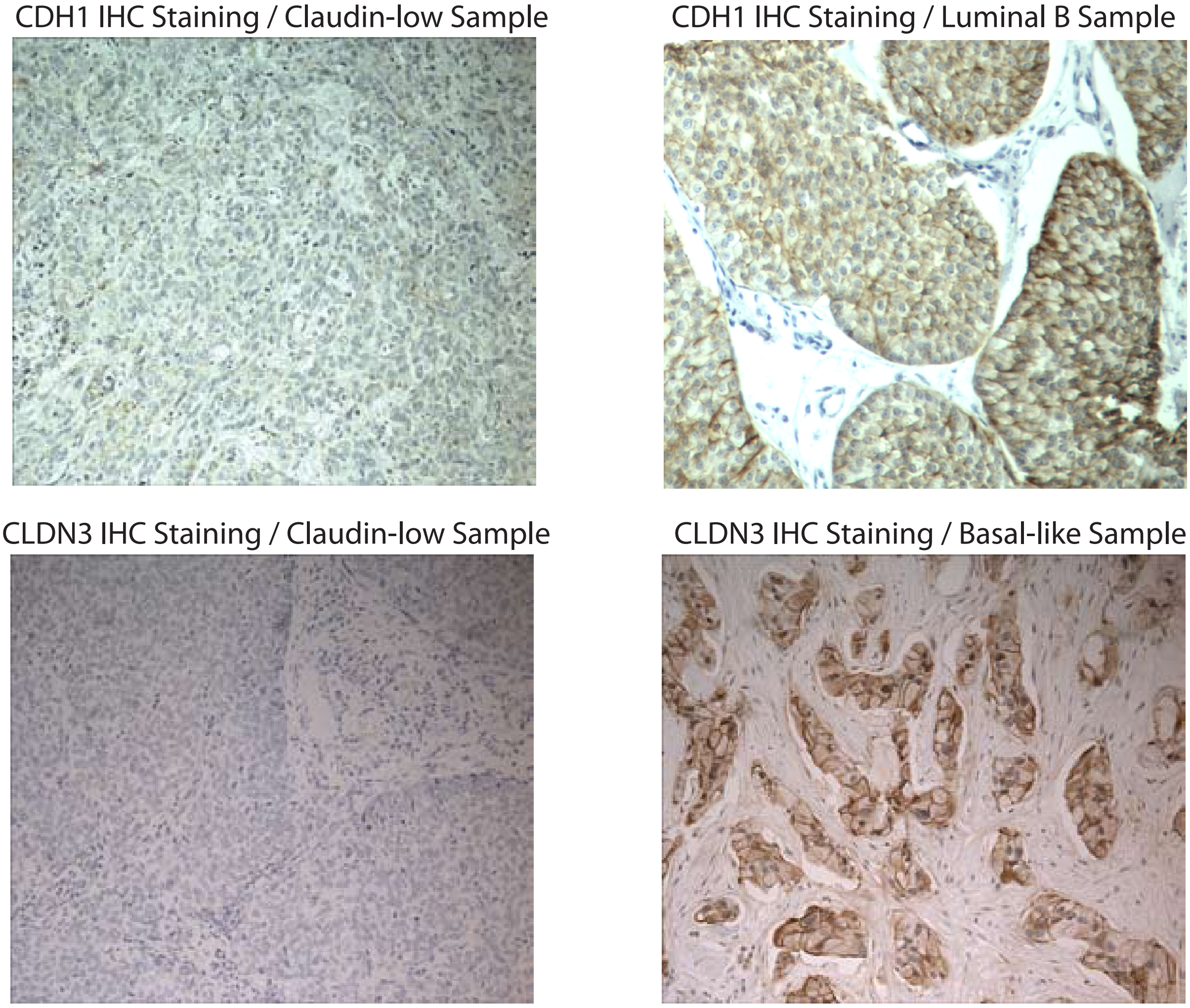

B

E-Cadherin (CDH1) and Claudin 3 (CLDN3) IHC Staining in 103 Breast Cancers.

Subtype	Samples with CDH1 Negative/Weak Positive Staining	%	Samples with CDH1 Moderate/Strong Positive Staining	%	Samples with CLDN3 Negative/Weak Positive Staining	%	Samples with CLDN3 Moderate/Strong Positive Staining	%	Total Samples
Claudin-low	10	45%	12	55%	13	59%	9	41%	22
Basal-like	2	11%	17	89%	2	11%	17	89%	19
HER2-enriched	1	10%	9	90%	5	50%	5	50%	10
Luminal A	4	16%	21	84%	7	28%	18	72%	25
Luminal B	5	22%	18	78%	4	17%	19	83%	23
Normal Breast-like	0	0%	4	100%	0	0%	4	100%	4
Total	22	21%	81	79%	31	30%	72	70%	103

CDH1 Statistics (Chi-Square Test)	P-value
Claudin-low vs rest	0.0019
Claudin-low vs Basal-like	0.0142

CLDN3 Statistics (Chi-Square Test)	P-value
Claudin-low vs rest	0.0008
Claudin-low vs Basal-like	0.0020

Figure S4

A

B

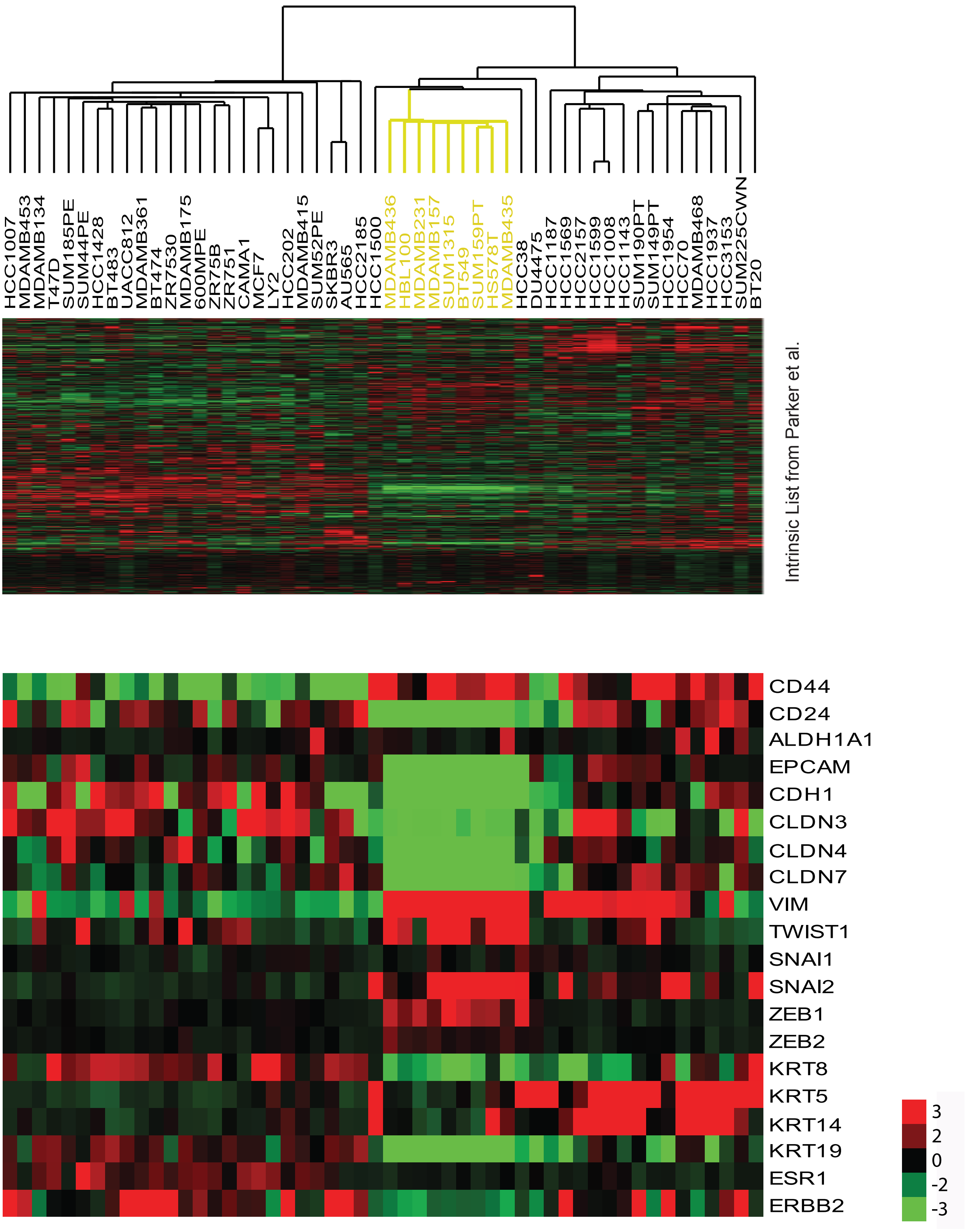

C

Claudin-low Breast Cancer Cell Lines		
BT549	MDA-MB157	MDA-MB436
HBL100	MDA-MB231	SUM159PT
Hs578T	MDA-MB435	SUM1315

Figure S5

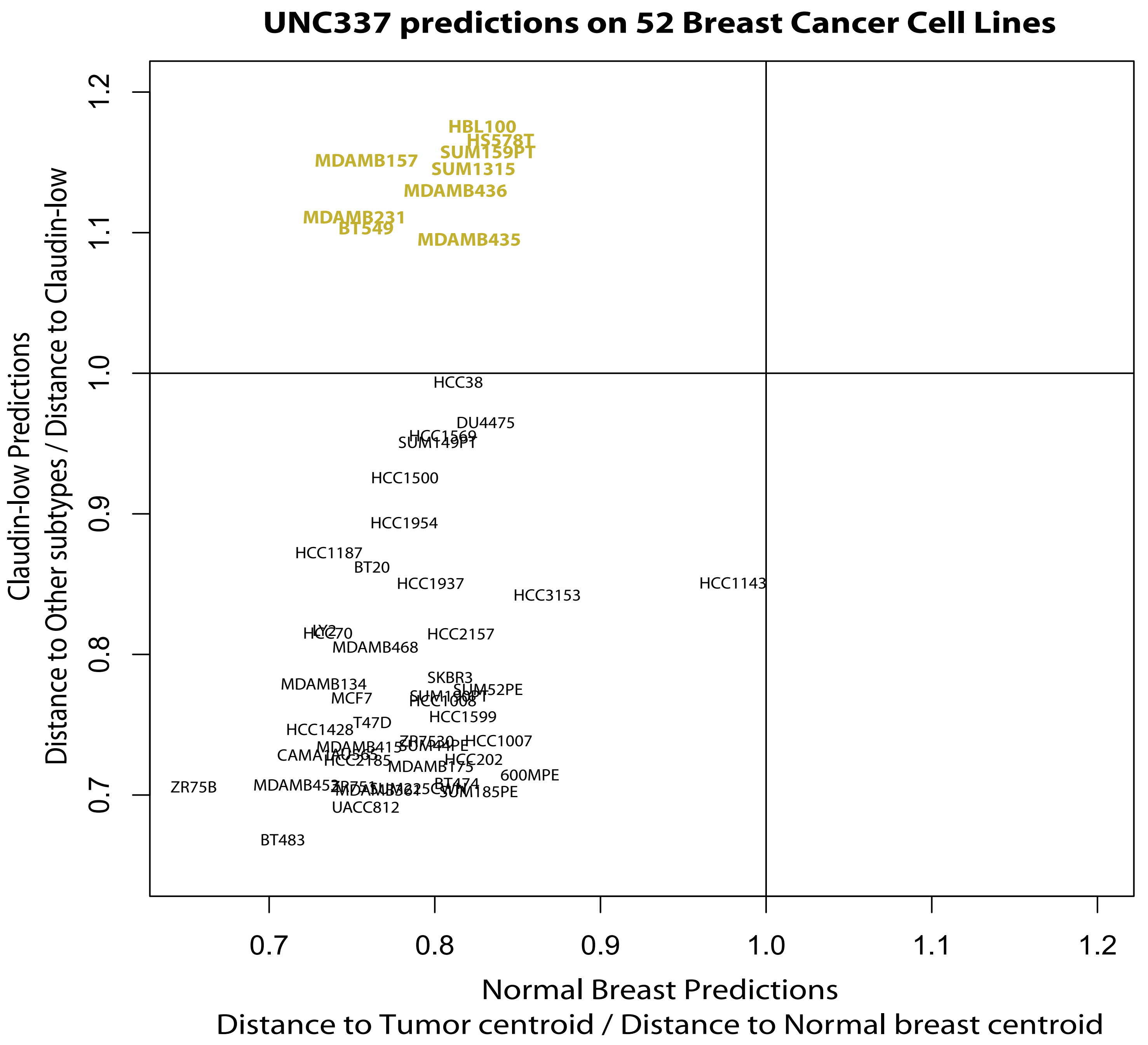

UNC337 predictions on 52 Breast Cancer Cell Lines
HBL100
HS578T
SUM159PT
MDAMB157
SUM1315
MDAMB436
MDAMB231
BT549
MDAMB435
HCC38
DU4475
HCC1569
SUM149PT
HCC1500
HCC1954
HCC1187
BT20
HCC1937
HCC3153
HCC1143
LY2
HCC70
HCC2157
MDAMB468
SKBR3
MDAMB134
SUM52PE
MCF7
HCC1008
HCC1599
T47D
HCC1428
HCC1007
CAMA1
HCC202
600MPE
ZR75B
BT474
SUM185PE
UACC812
BT483
0.7
0.8
0.9
1.0
1.1
1.2
Normal Breast Predictions
Distance to Tumor centroid / Distance to Normal breast centroid
Claudin-low Predictions
Distance to Other subtypes / Distance to Claudin-low

Figure S6

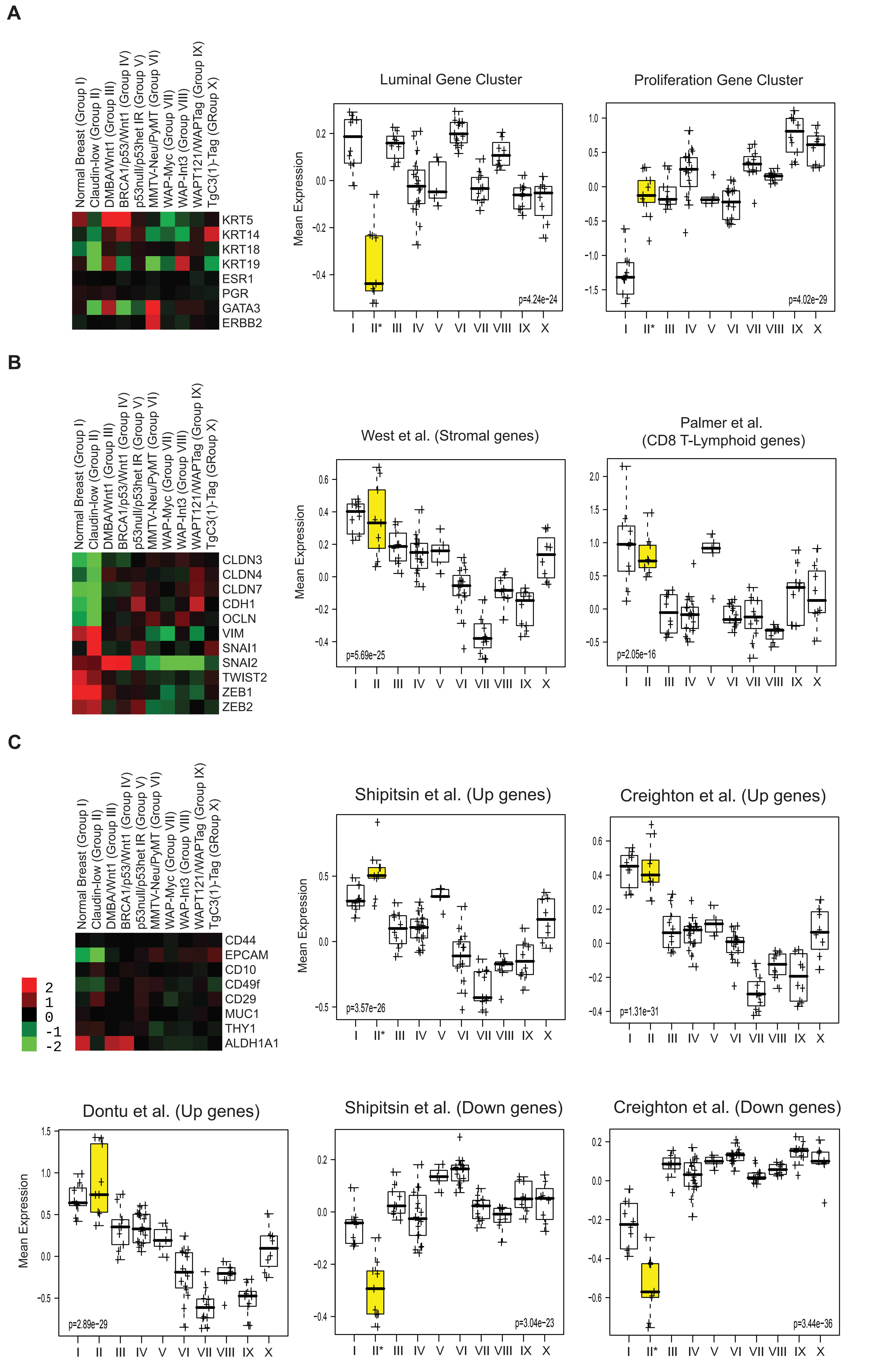

A
Normal Breast (Group I)
Claudin-low (Group II)
DMBA/Wnt1 (Group III)
BRCA1/p53/Wnt1 (Group IV)
p53null/p53het IR (Group V)
MMTV-Neu/PyMT (Group VI)
WAP-Myc (Group VII)
WAP-Int3 (Group VIII)
WAPT121/WAPTag (Group IX)
TgC3(1)-Tag (GRoup X)
KRT5
KRT14
KRT18
KRT19
ESR1
PGR
GATA3
ERBB2
Luminal Gene Cluster
Mean Expression
p=4.24e-24
I II* III IV V VI VII VIII IX X
Proliferation Gene Cluster
p=4.02e-29
I II* III IV V VI VII VIII IX X
B
CLDN3
CLDN4
CLDN7
CDH1
OCLN
VIM
SNAI1
SNAI2
TWIST2
ZEB1
ZEB2
West et al. (Stromal genes)
Mean Expression
p=5.69e-25
I II III IV V VI VII VIII IX X
Palmer et al.
(CD8 T-Lymphoid genes)
p=2.05e-16
I II III IV V VI VII VIII IX X
C
CD44
EPCAM
CD10
CD49f
CD29
MUC1
THY1
ALDH1A1
2
1
0
-1
-2
Shipitsin et al. (Up genes)
Mean Expression
p=3.57e-26
I II* III IV V VI VII VIII IX X
Creighton et al. (Up genes)
p=1.31e-31
I II III IV V VI VII VIII IX X
Dontu et al. (Up genes)
Mean Expression
p=2.89e-29
I II III IV V VI VII VIII IX X
Shipitsin et al. (Down genes)
p=3.04e-23
I II* III IV V VI VII VIII IX X
Creighton et al. (Down genes)
p=3.44e-36
I II* III IV V VI VII VIII IX X

Figure S7

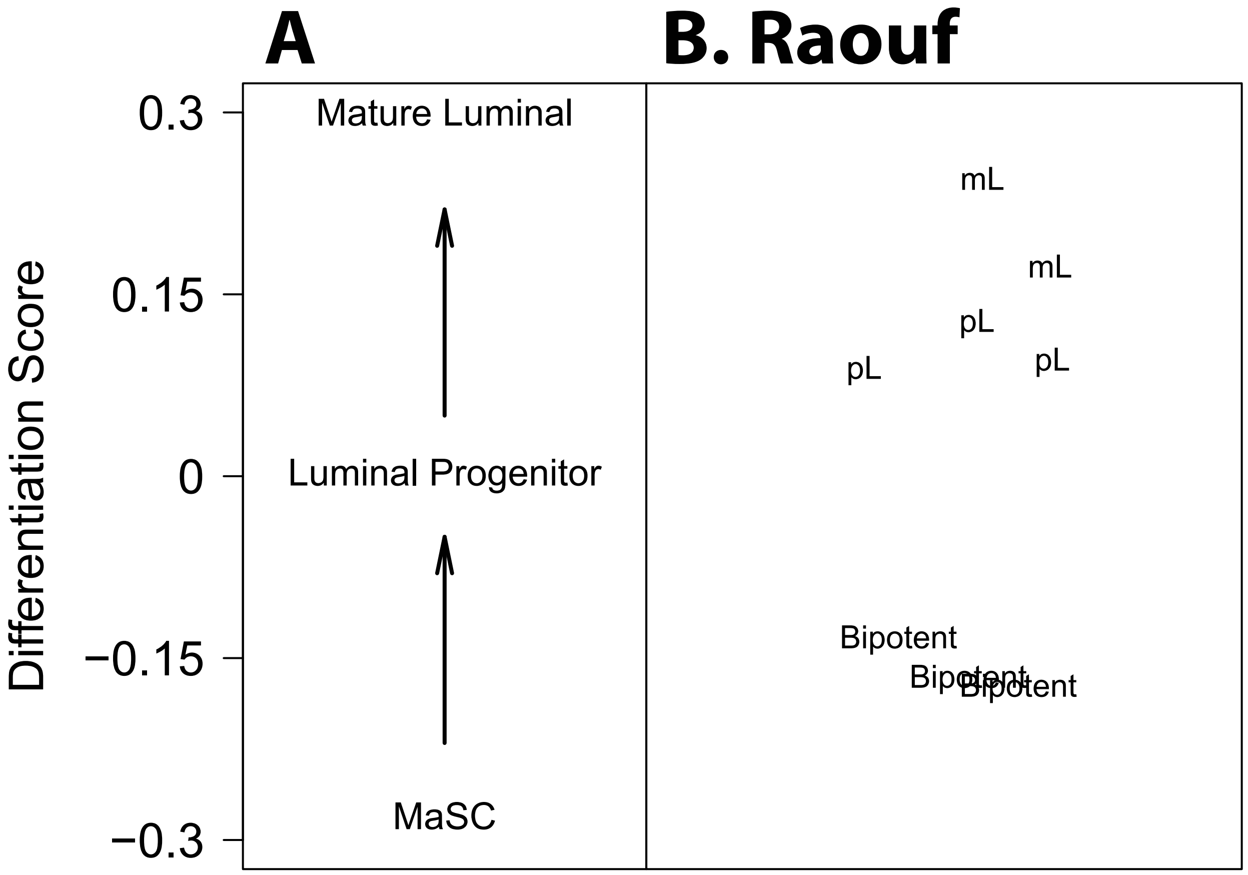
A
B. Raouf
Differentiation Score
0.3
0.15
0
−0.15
−0.3
Mature Luminal
Luminal Progenitor
MaSC
mL
mL
pL
pL
pL
Bipotent
Bipotent
Bipotent

Figure S8

9-Cell Line Claudin−low Predictor

A

Lim et al.

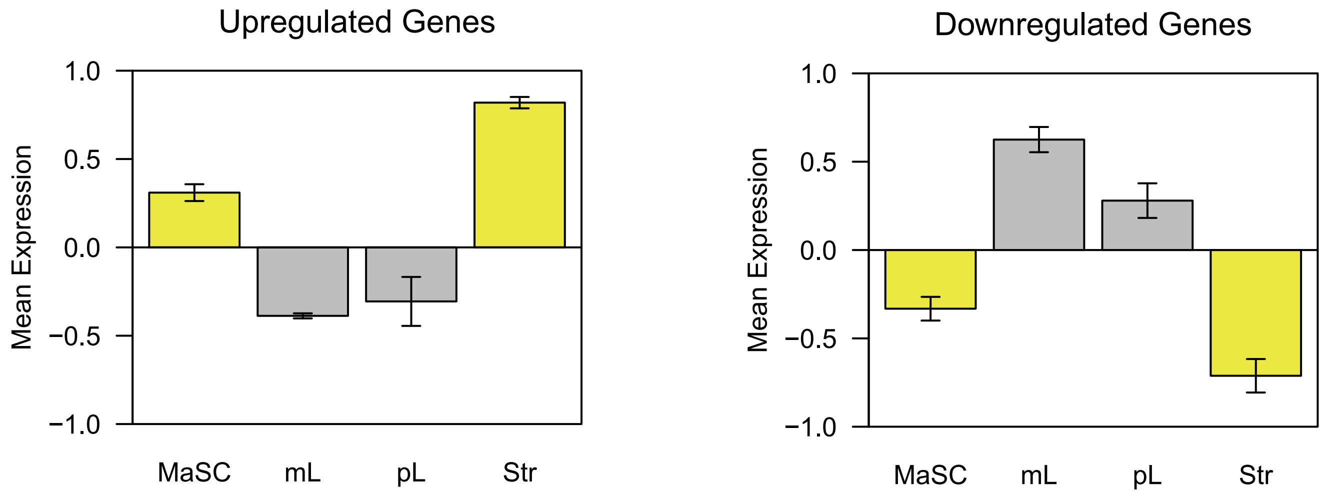

B

Raouf et al.

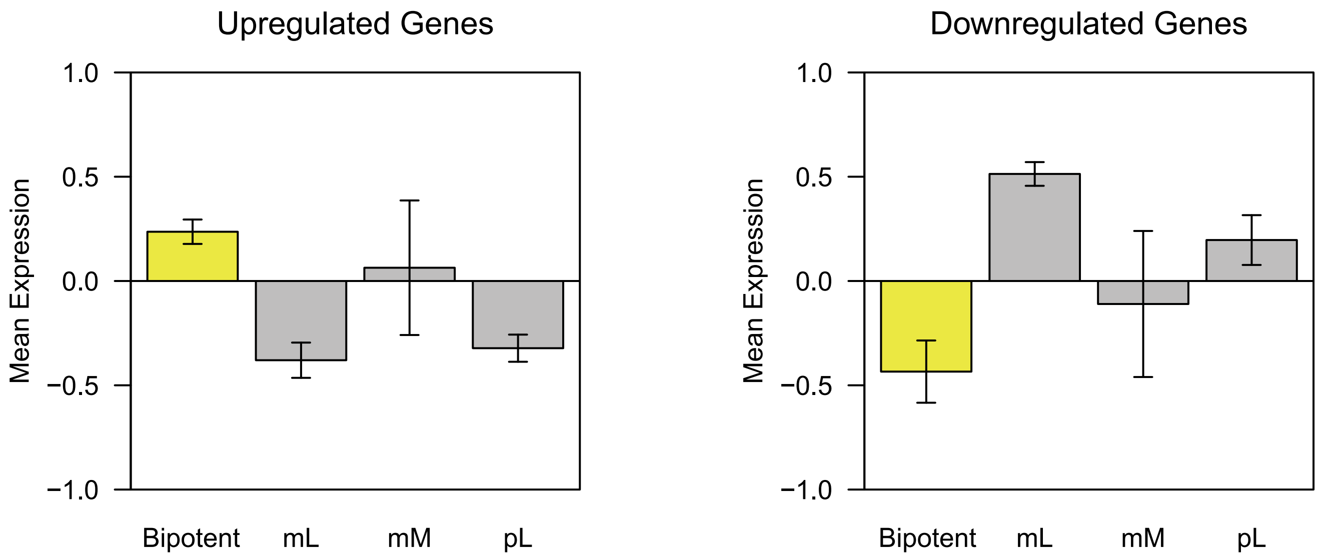

Figure S9

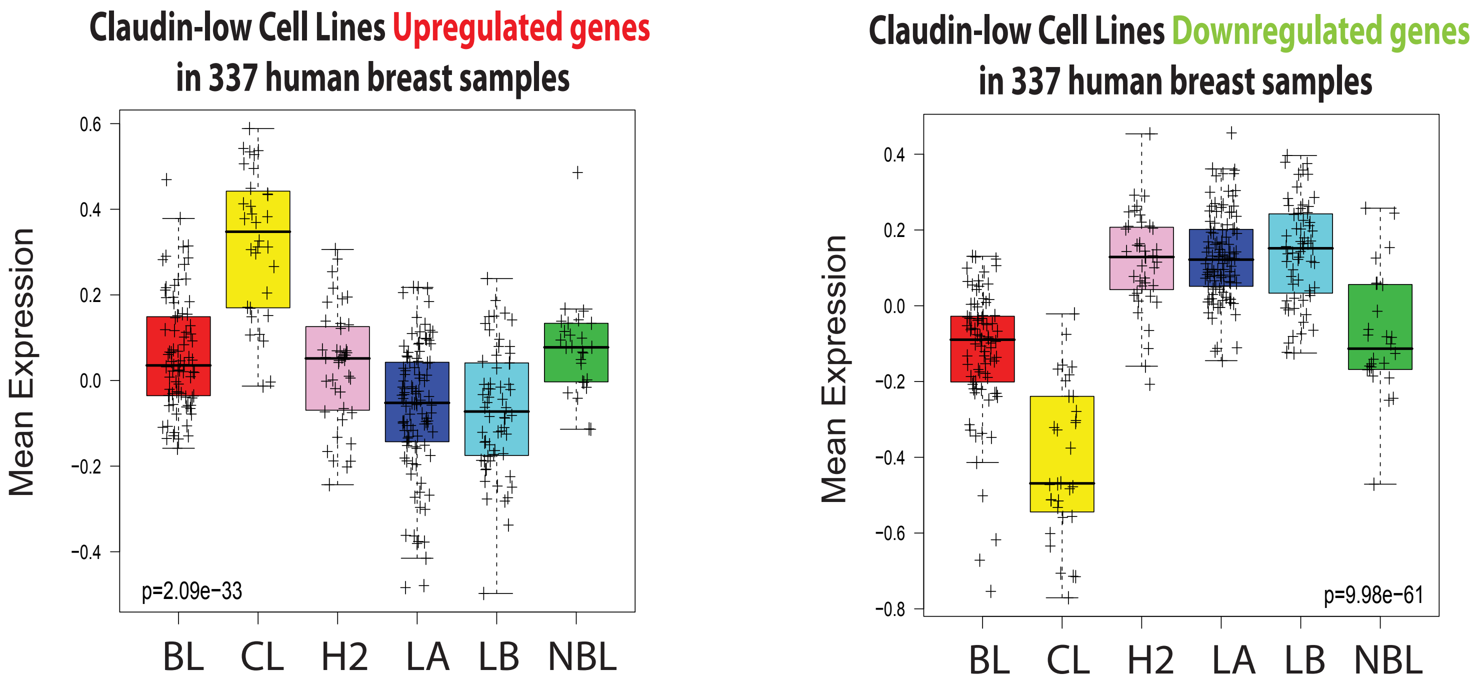
Claudin-low Cell Lines Upregulated genes
in 337 human breast samples
Mean Expression
0.6
0.4
0.2
0.0
-0.2
-0.4
p=2.09e−33
BL
CL
H2
LA
LB
NBL
Claudin-low Cell Lines Downregulated genes
in 337 human breast samples
Mean Expression
0.4
0.2
0.0
-0.2
-0.4
-0.6
-0.8
p=9.98e−61
BL
CL
H2
LA
LB
NBL

Figure S10

UNC337 Intrinsic Clustering as shown in **Figure S1**.
Yellow array cluster: Claudin-low cluster as defined by SigClust.
Red: the 5 Claudin-low H/E samples from Herschkowitz et al. Genome Biol 2007 (Supplemental Figure 7)

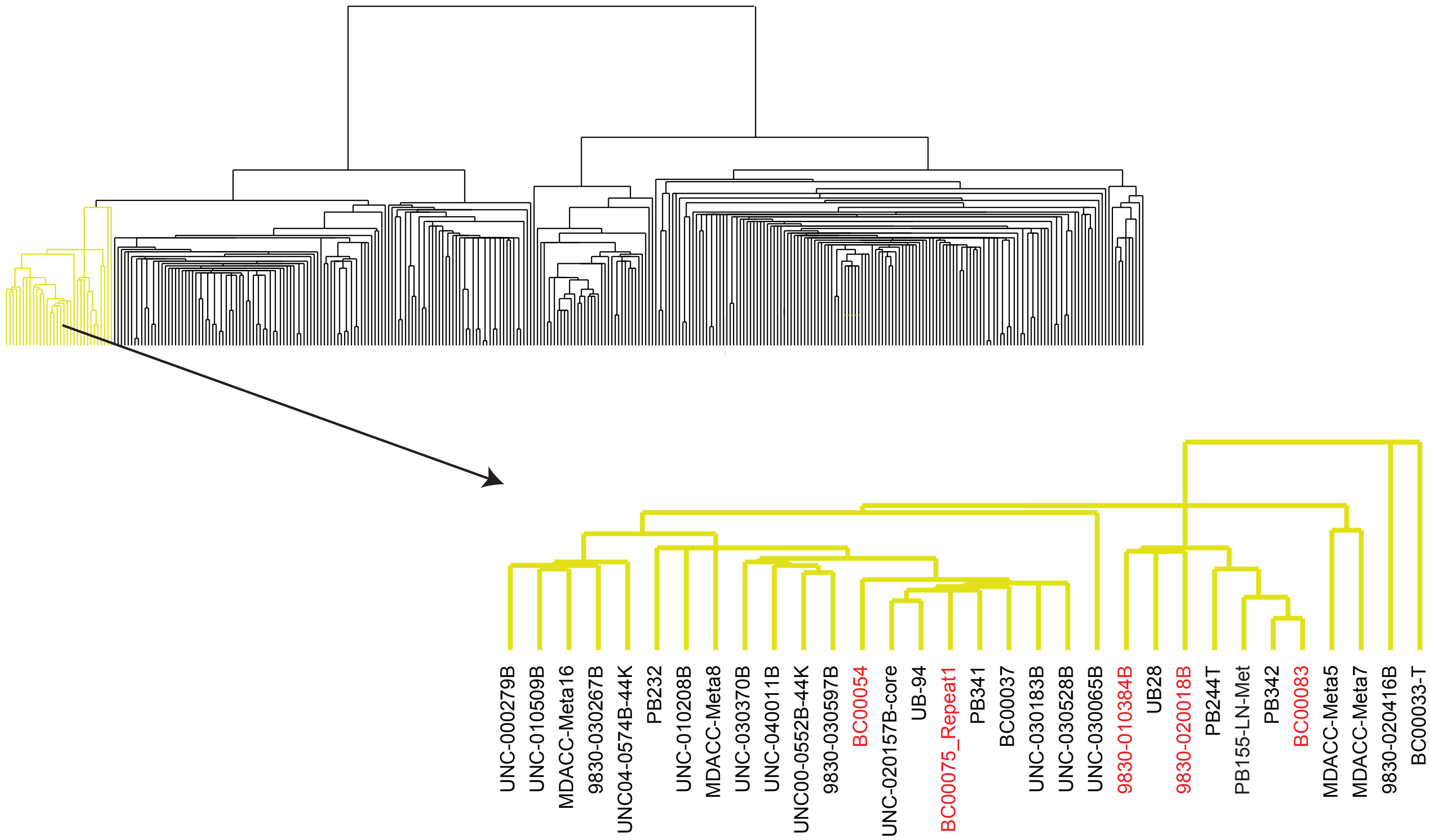

Supplement: Additional file 1 — Supplementary Tables S1-S5 and Supplementary Figures S1-S10. Table S1. Biological processes and signaling pathways enriched in claudin-low vs. basal-like tumors. Table S2. Biological processes and signaling pathways enriched in claudin-low tumors vs. rest. Table S3. Identification of the claudin-low subtype in a panel of breast cancer cell lines. Table S4. Histological examination of claudin-low tumors. Table S5. Evaluation of the intrinsic breast cancer molecular subtypes in histologically diverse types. Figure S1. Intrinsic unsupervised hierarchical clustering of the UNC337 database. Figure S2. Average expression of additional selected genes and gene signatures across the breast cancer subtypes. Figure S3. E-cadherin and claudin 3 immunohistochemical staining of breast tumors. Figure S4. Intrinsic gene set analysis of 52 breast cancer cell lines. Figure S5. Claudin-low tumor and normal breast predictions in 52 breast cancer cell lines. Figure S6. Average expression of genes and gene signatures across the various mouse classes. Figure S7. Differentiation predictions in Raouf et al. [23] database. Figure S8. Expression of the nine-cell line claudin-low predictor across different subpopulations of the normal breast. Figure S9. Mean expression of the top highly expressed (n = 833) and low expressed (n = 642) genes in claudin-low cell lines across 337 human breast tumor samples classified according to intrinsic subtype, including the normal breast-like group. Figure S10. Localization of five claudin-low samples (BC00054, 020018B, BC00075, 010384B, and BC00083) in the UNC337 intrinsic clustering. [file bcr2635-S1.PDF]
